# First-aid, pre-hospital care, and harmful indigenous practices in pediatric snakebite envenomation: A systematic review of global evidence from 1973 to 2025

**DOI:** 10.1371/journal.pntd.0014508

**Published:** 2026-07-28

**Authors:** Nayana Gunathilaka, Iranga Dilshan, Kaumudee Kodikara, Kavinda Dayasiri

**Affiliations:** 1 Department of Parasitology, Faculty of Medicine, University of Kelaniya, Ragama, Sri Lanka; 2 Department of Medical Education, Faculty of Medicine, University of Kelaniya, Ragama, Sri Lanka; 3 Department of Paediatrics, Faculty of Medicine, University of Kelaniya, Ragama, Sri Lanka; Griffith University - GC Campus: Griffith University - Gold Coast Campus, AUSTRALIA

## Abstract

**Background:**

Snakebite envenomation is a neglected tropical disease that disproportionately affects children in low- and middle-income countries. Pediatric patients are uniquely vulnerable due to smaller body mass, increased venom-to-body-weight ratios, and behavioral risk factors such as outdoor play. The pre-hospital phase is critical in determining outcomes, yet inappropriate first-aid and harmful indigenous practices often characterize it. Despite the recognized importance of early interventions, global evidence on pediatric-specific pre-hospital care remains fragmented. This systematic review synthesizes available literature on first-aid measures, pre-hospital interventions, and harmful traditional practices in pediatric snakebite envenomation.

**Method:**

Following PRISMA 2020 guidelines, a comprehensive search was conducted in PubMed/MEDLINE, Scopus, and Lens.org, covering publications from 1973 to December 2025. Eligible studies included children and adolescents (0–18 years) with snakebite envenomation, reporting first-aid or pre-hospital practices and associated outcomes. Observational, interventional, and qualitative designs were included, while case reports, reviews, and non-English publications were excluded. Risk of bias was assessed using the Newcastle–Ottawa Scale, Joanna Briggs Institute checklist, and CASP qualitative tool. Due to heterogeneity, findings were synthesized narratively.

**Results:**

Forty-four studies were included, representing South Asia, Sub-Saharan Africa, Latin America, the Middle East, Europe, and North America. Harmful practices were most prevalent in South Asia and Africa, where tourniquet use ranged from 38-78%, incision and suction from 22-64%, and herbal remedies from 28-58%. Consultation with traditional healers was frequent, reported in 40–73% of cases. Median delays in hospital presentation were longest in Africa and South Asia, often exceeding 6–18 hours, and each hour of delay increased severity odds by 1.4‑fold. Mortality varied regionally, from 0.06% in North America to 8–10% in Africa, with inappropriate first‑aid identified as an independent predictor of poor outcomes. Knowledge gaps were consistent across regions, with fewer than 20% of parents able to identify appropriate first‑aid and 67% of healthcare providers reporting inadequate training.

**Conclusion:**

This review demonstrates that harmful indigenous practices remain pervasive in pediatric snakebite management worldwide, contributing to preventable morbidity and mortality. Evidence underscores the urgent need for standardized, evidence-based pre-hospital guidelines, culturally sensitive community education, and targeted training for healthcare providers. Addressing these gaps through policy development and educational interventions is essential to reduce the global burden of pediatric snakebite envenomation.

## Author summary

Snakebite envenomation is a life-threatening neglected tropical disease that disproportionately affects children in low- and middle-income countries across tropical and subtropical regions. Children face unique biological vulnerabilities, including higher venom-to-body-mass ratios and narrower limb circumferences, making them especially susceptible to severe outcomes. Yet the critical period between the bite and hospital arrival, when families and communities must act, is shaped not by medical guidelines but by cultural beliefs, fear, and limited health literacy. In this systematic review, we synthesized evidence from 44 studies published between 1973 and 2025 to understand what first-aid and pre- hospital practices are actually being applied to children with snakebite. Our findings reveal that harmful practices such as tourniquet application, incision and suction of bite wounds, herbal poultices, and consultation with traditional healers are pervasive across South Asia, sub-Saharan Africa, Latin America, and the Middle East. These practices delay hospital presentation and independently predict poor outcomes, including death. Fewer than 20% of parents could identify appropriate first aid. This review underscores an urgent need for culturally sensitive education, standardized pediatric first-aid guidelines, and targeted training of healthcare providers to prevent avoidable death and disability in children affected by snakebite.

## Introduction

Snakebite envenomation (SBE) among children is a major but neglected public health problem, especially in low‑ and middle‑income countries in the tropics and the subtropics [1]. The World Health Organization (WHO) lists SBE as a category A neglected tropical disease. Each year, an estimated 1.8--2.7 million envenomations occur globally, leading to 81,000--138,000 deaths and causing long‑term disability and socioeconomic burden [2]. Children are particularly vulnerable, representing a significant proportion of cases in endemic regions and often experiencing more severe outcomes than adults [3,4].

Several factors explain this vulnerability. Children have lower body weight, which results in a higher venom‑to‑body‑mass ratio and faster systemic toxicity [5,6]. Their curiosity, limited awareness of environmental risks, and frequent outdoor play increase exposure to venomous snakes [7]. Anatomical features, such as a smaller limb circumferencemean that the same amount of venom can cause more extensive local damage and higher compartment pressures in children compared to adults [8]. Socioeconomic and geographic factors exacerbate the risk, as most pediatric snakebites occur in rural farming communities with limited access to healthcare, inadequate education, and a strong influence of traditional beliefs [9–11]. The period between the bite and hospital treatment is critical but poorly standardized. First‑aid is often provided by family members, neighbors, or traditional healers, guided more by cultural beliefs and traditions than by evidence‑based practice [11–13]. Pathways to care are shaped by fear, low health literacy, geographic barriers, and reliance on traditional practices [11,14,15].

Harmful indigenous practices are common. These include the use of tight tourniquets, which can cause ischemia and compartment syndrome, incision and suction of bite sites that can lead to infection, application of ice or herbal poultices that delays care, and the use of traditional remedies with toxic effects or that interfere with medical treatment [12,16]. In many endemic areas, consultation with traditional healers occurs before or instead of hospital care, delaying antivenom administration and supportive treatment [10,11,15], These harmful practices persist due to limited health education in schools and universities [17], mistrust of modern healthcare and financial barriers [11,18–22], and the authority of traditional healers [11].

Although the importance of pre‑hospital care is well recognized, it is important to clarify the terminology used in this review. First aid refers to the immediate assistance given to a snakebite victim before professional medical treatment is available, including measures such as immobilization, wound care, and decisions about transport. Pre-hospital care is a broader concept encompassing all care delivered outside the formal healthcare setting, including both first aid and any interventions by community members, traditional healers, or first responders prior to hospital admission. These two terms are used distinctly throughout this review and are not treated as synonyms. Despite this recognized importance, significant gaps remain in the literature. These gaps include: (i) a lack of synthesis of pediatric-specific evidence on first-aid and pre-hospital practices, distinct from adult populations; (ii) limited understanding of the prevalence and regional variation in harmful indigenous practices in children; (iii) poorly defined training needs of healthcare providers in resource-limited settings [23]; (iv) inadequate data on parental knowledge, attitudes, and health-seeking behavior during pediatric snakebite emergencies; and (v) an absence of rigorous evidence on the effectiveness of community education and behavioral interventions. Most studies describe epidemiology and hospital management of pediatric snakebite [23–25], but few synthesize evidence on first-aid and harmful practices. The training needs of healthcare providers in resource-limited settings are also poorly defined [15,26]. Understanding the range of first-aid practices, the factors that sustain harmful interventions, and the barriers to evidence-based care is essential for designing effective education programs and improving outcomes.

This systematic review aims to synthesize evidence on first‑aid practices, pre‑hospital care, and harmful indigenous practices in pediatric snakebite envenomation. It seeks to identify common interventions across regions, assess the prevalence and types of harmful practices, and evaluate their impact on outcomes. The review also examines parental knowledge, attitudes, and decision-making in relation to first aid and health-seeking behaviors. Ultimately, it highlights knowledge gaps and offers recommendations for future research, policy, and community education.

## Method

### Protocol and registration

This review was conducted and reported in accordance with the PRISMA 2020 guidelines. The protocol was not registered prospectively in PROSPERO or any comparable registry, and this is acknowledged as a limitation. Our decision to undertake the review emerged from preliminary scoping work, which identified a notable gap: no existing synthesis had examined pre-hospital snakebite management specifically in the pediatric age group. To guard against post hoc methodological choices, eligibility criteria, data extraction procedures, and planned analyses were specified in an internal protocol before the database searches were run, and none of these decisions were altered after searching commenced. Readers are encouraged to keep this limitation in mind when weighing the findings, given that prospective registration is the accepted standard for limiting selective outcome reporting (S1 Fig).

### Eligibility criteria

Studies were eligible if they involved children and adolescents (0–18 years) with snakebite envenomation and reported on first-aid or pre-hospital practices, including both appropriate and potentially harmful interventions, such as tourniquet use, incision and suction, herbal remedies, and consultations with traditional healers. Outcomes of interest included morbidity, mortality, complications, delays in care, appropriateness of first-aid, and healthcare-seeking behaviors, with secondary outcomes addressing knowledge, attitudes, and provider perspectives. Eligible designs included observational, interventional, and qualitative studies written in English. Single-case reports, reviews, editorials, conference abstracts, and non-English publications were excluded. Restricting eligibility to English-language publications was a pragmatic decision driven by the language expertise available within the research team; we recognise that this likely introduced some degree of language bias. Important literature from Latin America, sub-Saharan Africa, and South and Southeast Asia may consequently have been missed, and readers should bear this in mind when assessing the geographic scope of the evidence. Studies restricted to adult populations, those reporting only in-hospital management without a pre-hospital component, animal or laboratory research, duplicate cohorts, and papers with insufficiently described methods were all excluded.

### Information sources

A comprehensive literature search was conducted across three major electronic databases: PubMed/MEDLINE, Scopus, and Lens.org. These databases were selected to ensure broad coverage of medical, scientific, and interdisciplinary literature relevant to snakebite envenomation. Trial registries and grey literature sources were not searched, and we consider this a meaningful limitation. Unpublished hospital audits, national health ministry reports, and program evaluations from high-burden countries could hold relevant data on community-level pre-hospital practices that simply do not appear in peer-reviewed journals. Reference list screening was not applied systematically, though pertinent citations encountered during full-text review were recorded and considered for inclusion.

### Search strategy

The search strategy was developed iteratively with input from the research team and incorporated both Medical Subject Headings (MeSH) terms and free-text keywords. The strategy combined three main concept groups using Boolean operators: snakebite-related terms, first-aid and pre-hospital care terms, and pediatric population terms.

For PubMed/MEDLINE, the search utilized MeSH terms including “Snake Bites”[Mesh], “Snakes”[Mesh], “First Aid”[Mesh], “Emergency Medical Services”[Mesh], “Child”[Mesh], “Infant”[Mesh], and “Adolescent”[Mesh], combined with text words such as snakebite*, pre-hospital*, prehospital*, first-aid, traditional practice*, indigenous practice*, harmful practice*, child*, pediatric*, paediatric*, and related variants. For Scopus and Lens.org, adapted search strategies using TITLE-ABS-KEY fields and field-specific operators were employed to maintain consistency while accounting for database-specific syntax requirements.

### Study selection

All retrieved records were imported into reference management software, and duplicate records were identified and removed using both automated and manual methods. The study selection process was conducted in two stages by two independent reviewers (initials blinded for review). In the first stage, titles and abstracts of all unique records were screened against the predefined eligibility criteria. Records that clearly did not meet the inclusion criteria were excluded at this stage. In cases of uncertainty, studies were advanced to full-text review. In the second stage, full-text articles of potentially eligible studies were retrieved and assessed independently by two reviewers against the complete set of inclusion and exclusion criteria. Disagreements between reviewers at both stages were resolved through discussion, and when consensus could not be reached, a third reviewer was consulted.

**Fig 1:**
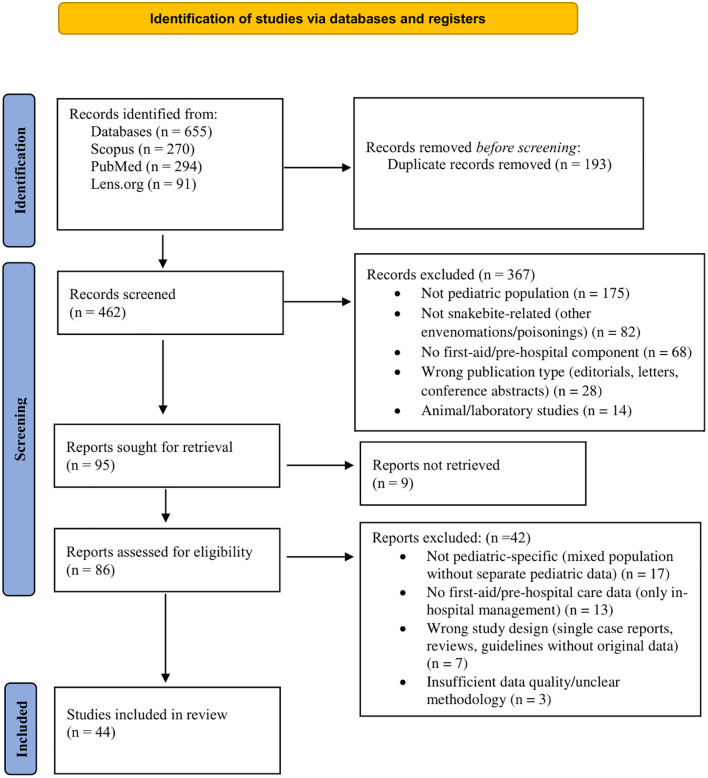
PRISMA flow diagram illustrating the article selection and screening.

The study selection process and reasons for exclusion at each stage are documented in the PRISMA 2020 flow diagram (Fig 1). Reasons for exclusion of full-text articles were systematically recorded and categorized into not pediatric-specific, no first-aid or pre-hospital care data, wrong study design, insufficient data quality or unclear methodology, and duplicate data from the same cohort (S1 Table).

### Data extraction process

Data from the included studies were systematically extracted by two independent reviewers using a standardized, pilot-tested Excel sheet that captured study and population characteristics, details of first-aid and pre-hospital practices, clinical outcomes, and methodological features. Variables recorded included demographics, snake species, severity of envenomation, types and prevalence of first-aid or harmful practices, delays in care, and healthcare-seeking behaviors, alongside outcomes such as complications, disability, and mortality. Methodological aspects, including diagnostic criteria, outcome measures, and potential biases, were also documented. Discrepancies were resolved through discussion, with attempts made to clarify missing or unclear data from the study authors; however, this was not always successful for older publications (S2 Table).

### Risk of bias and quality assessment

The methodological quality and risk of bias of included studies were assessed independently by two reviewers using tools appropriate to each study design. For observational studies including cross-sectional, case-control, and cohort studies, the Newcastle–Ottawa Scale (NOS) was employed, which evaluates selection of study groups, comparability of groups, and ascertainment of exposure or outcome. For case series and descriptive studies without comparison groups, the Joanna Briggs Institute (JBI) Critical Appraisal Checklist for Case Series was utilized. Qualitative studies were assessed using the Critical Appraisal Skills Programme (CASP) Qualitative Checklist, which evaluates rigor, credibility, and relevance.

Each study was classified as having a low, moderate, or high risk of bias based on its overall assessment across the various domains. Disagreements in quality assessment were resolved through discussion, with arbitration by a third reviewer when necessary. The quality assessment results were used to contextualize findings during synthesis. Still, they were not used as exclusion criteria, as excluding studies based on quality might have resulted in loss of valuable information from resource-limited settings where methodological rigor may be constrained (S3 Table).

### Data synthesis

Due to substantial heterogeneity in study designs, populations, interventions, outcomes, and measurement methods, a narrative synthesis was conducted instead of a meta-analysis. Findings were organized thematically around first-aid practices, harmful pre-hospital interventions, traditional healing methods, knowledge and attitudes, healthcare-seeking behaviors, and clinical outcomes. Data were described by region, practice type, and population characteristics, with subgroup analyses by geography, practice category, and setting. The strength of evidence was assessed qualitatively; however, quantitative pooling was avoided due to the diverse outcome measures, inconsistent reporting, variation in snake species, and differences in healthcare systems, which rendered meta-analysis inappropriate.

## Results

### Study selection

The systematic search identified 1,310 records, of which 193 duplicates were removed, resulting in 1,117 records for screening. After title and abstract review, 367 records were excluded for reasons such as non-pediatric focus, unrelated topics, lack of pre-hospital data, inappropriate publication type, or animal studies. Ninety-three reports were sought for full-text review, but nine could not be retrieved. Of the 84 assessed, 42 were excluded due to mixed populations lacking pediatric data, a focus on in-hospital care only, an unsuitable study design, poor data quality, or duplication. Ultimately, 44 studies met all inclusion criteria and were incorporated into the systematic review, as detailed in the PRISMA flow diagram (Fig 1).

### Characteristics of included studies

**Fig 2:**
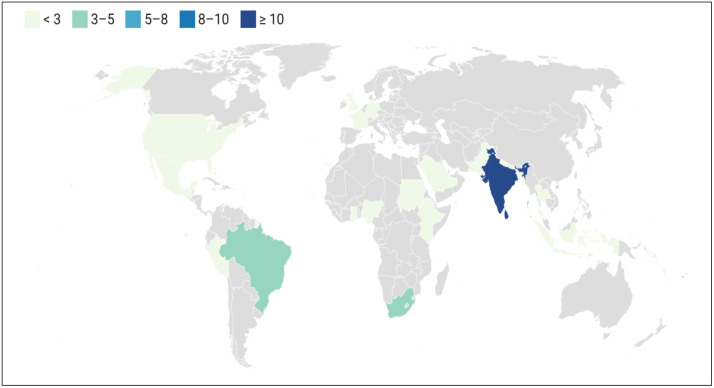
Geographic distribution of studies included in the systematic review. Base map sourced from Natural Earth (http://www.naturalearthdata.com), public domain. Natural Earth data are free to use without restriction and are fully compatible with the CC BY 4.0 license.

The 44 included studies were published between 1973 and 2025, with a notable increase in publication frequency observed after 2015, reflecting growing recognition of pediatric snakebite as a distinct clinical entity requiring specialized attention. Geographically, the included studies represented diverse snakebite-endemic regions, with the majority conducted in South Asia (particularly India, Sri Lanka, and Bangladesh), Sub-Saharan Africa (Nigeria, South Africa, Sudan), the Middle East (Saudi Arabia, Israel), South America (Brazil), and smaller numbers from Southeast Asia, Europe, and North America. This geographic distribution aligns with the global burden of pediatric snakebite envenomation in low- and middle-income countries, particularly in tropical and subtropical regions. The geographic distribution of included studies is illustrated in Fig 2.

Study designs varied considerably across the included literature. The majority were retrospective cohort studies (n = 18) and prospective observational studies (n = 14), with additional contributions from cross-sectional surveys (n = 7), case series (n = 3), and mixed-methods studies (n = 2). Sample sizes ranged from small case series of fewer than 20 patients to large multicenter cohorts comprising more than 500 pediatric snakebite cases. The age range of the included populations generally encompassed children from birth through 18 years, although some studies focused specifically on younger age groups (under 5 years or under 12 years), while others included adolescents up to 15 or 16 years, based on local definitions of pediatric populations.

Study settings demonstrated considerable heterogeneity. Hospital-based studies examining pre-hospital practices through retrospective case note review or structured interviews with caregivers at the time of hospital presentation predominated (n = 28). Community-based studies employing household surveys, interviews with parents, and assessments of knowledge and practices in snakebite-endemic villages provided complementary perspectives (n = 12). Several studies employed mixed approaches, combining hospital surveillance data with community outreach and educational assessments (n = 4). Detailed characteristics of all included studies are presented in Table 1.

**Table 1:** Characteristics of included studies in the systematic review.

Author(s), Year	Country	Study Design	Sample Size	Age Group	Type of First-aid/Pre-hospital Practices Assessed	Harmful Indigenous Practices Reported	Key Outcomes Related to Pre-hospital Care
Geyt et al., 2020 [1]	Multi-country review	Narrative review with case examples	N/A	Pediatric focus	WHO guidelines, tourniquet, immobilization, rapid transport	Tourniquet, incision, suction identified as harmful	Recommended abandonment of tourniquet and incision
Oliveira et al., 2023 [3]	Brazil	Retrospective cohort	345	0-15 years	Tourniquet, incision, traditional remedies, electric shock, time to hospital	Tourniquet (45%), incision (23%), traditional remedies (31%), electric shock (8%)	Tourniquet use associated with increased local complications (OR 2.8)
Variawa et al., 2020 [4]	South Africa	Prospective review	89	0-13 years	Pre-hospital interventions, tourniquet complications	Tourniquet (41%), traditional healer (18%)	Tourniquet-related compartment syndrome in 12%
Sankar et al., 2013 [5]	India	Prospective observational	212	1-12 years	First-aid, time to hospital, predictors of outcome	Tourniquet (54%), traditional medicine (38%), incision (22%)	Each hour delay increased severity odds by 1.4-fold; tourniquet associated with complications
Suryanarayana et al., 2020 [6]	India	Prospective cohort	167	1-15 years	First-aid practices, time to hospital, risk factors for poor outcomes	Tourniquet (62%), incision (34%), traditional medicine (28%)	Delay >6 hours independent predictor of poor outcome (OR 3.4)
Dayasiri et al., 2025e [7]	Sri Lanka	Mixed-methods study	420 parents	Children 0–18 years	Parental beliefs, traditional practices, emergency response patterns	Traditional healer consultation (52%), spiritual causation beliefs (38%)	Fear and traditional beliefs delayed appropriate care in 64%
Mars et al., 1991 [8]	South Africa	Case series with compartment pressure monitoring	8	4-13 years	Tourniquet use, compartment syndrome, surgical intervention	Tourniquet in all cases	Direct compartment pressure measurement guided management; 3 required fasciotomy
Dayasiri et al., 2025d [9]	Sri Lanka	Cross-sectional household survey	384 parents	Children 0–15 years	Preventive practices, parental knowledge, attitudes toward snakebite	Traditional healer belief in 41%, inadequate first-aid knowledge in 76%	Only 18% of parents could identify appropriate first-aid
Ahmed et al., 2019 [10]	Sudan	Descriptive cross-sectional	186	1-15 years	Traditional practices, clinical presentations, management	Traditional healer (68%), cauterization (34%), incision (52%), herbal remedies (58%)	High mortality (8.6%); traditional practices caused additional burns and infections
Nduagubam et al., 2020 [12]	Nigeria	Cross-sectional hospital-based	98	2-15 years	Comparison of practices with WHO guidelines	Tourniquet (71%), incision (58%), traditional medicine (44%), oral suction (31%)	89% received at least one harmful first-aid; none received WHO-recommended care
Dayasiri et al., 2025a [13]	Sri Lanka	Cross-sectional survey	462	0-18 years	Tourniquet application, immobilization, incision, traditional medicine, health-seeking behavior	Tourniquet (78%), incision (12%), traditional medicine application (34%)	Inappropriate first-aid in 82% of cases; delay >6 hours in 45%
Cristino et al., 2025 [14]	Brazil	Qualitative thematic drawing-and-story study	30	6-12 years	Therapeutic itineraries, traditional healer consultation, herbal remedies, delay in seeking care	Traditional healer consultation (63%), herbal poultices (47%), oral traditional remedies (23%)	Median delay 8 hours; traditional healer consultation associated with delayed hospital presentation
Dayasiri et al., 2025b [15]	Sri Lanka	Cross-sectional survey of healthcare providers	156 providers	N/A (pediatric cases managed)	Training needs assessment, knowledge of pediatric-specific management	Healthcare providers reported inadequate training in 67%	Identified gaps in pre-hospital triage and first-aid counseling
Bush & Kinlaw, 2015 [16]	USA	Case report with management review	1	8 years	Tight tourniquet complications, surgical management	Prolonged tight tourniquet (24 hours)	Severe limb ischemia requiring emergency surgical intervention
Tadros et al., 2022 [23]	USA	Retrospective ED database analysis	8,567	0-17 years	Emergency department visit patterns, pre-hospital care utilization	Limited data on pre-hospital practices in ED records	Most received appropriate pre-hospital care; low complication rate
Schulte et al., 2016 [24]	USA	Retrospective national database	25,770	0-19 years	Epidemiology, hospitalization, outcomes	Tourniquet (2.3%), incision (0.8%) - low rates in high-resource setting	Mortality 0.06%; inappropriate first-aid rare but associated with complications
Buitendag et al., 2021b [25]	South Africa	Comparative cohort study	156 (78 pediatric)	0-16 years vs adults	Tourniquet, traditional medicine, time to presentation	Tourniquet (38%), traditional medicine (29%)	Children had worse outcomes; pre-hospital delay >4 hours associated with mortality
Dayasiri et al., 2025c [26]	Sri Lanka	Qualitative interviews with physicians	28 physicians	N/A (pediatric cases managed)	Physician perspectives on pre-hospital practices, challenges in management	Tourniquet complications observed in 34% of severe cases	Pre-hospital tourniquet use identified as major challenge
Giri et al., 2020 [27]	India	Prospective observational PICU study	52	1-12 years	First-aid at home, time to hospital, PICU outcomes	Tourniquet (67%), traditional medicine (35%)	Mortality 11.5%; delay and inappropriate first-aid associated with PICU admission
Sood et al., 2020 [28]	India	Retrospective cohort	134	1-15 years	Epidemiology, first-aid practices, clinical profile	Tourniquet (58%), traditional remedies (42%), incision (28%)	Local complications more common with tourniquet use
Pandey et al., 2020 [29]	Nepal	Cross-sectional school survey	422 students	10-18 years	Student perceptions, knowledge of snakes and snakebite first-aid	Traditional beliefs about snake killing (67%), inadequate first-aid knowledge (78%)	Educational intervention improved knowledge scores
Sanni et al., 2021 [30]	Nigeria	Prospective cohort	67	1-14 years	First-aid, traditional healer use, outcomes	Traditional healer (73%), incision (64%), herbal application (52%)	Mortality 10.4%; traditional healer use associated with delayed presentation (mean 18 hours)
Pattanaik et al., 2023 [31]	India	Retrospective cohort	142	1-15 years	Clinical profile, first-aid, acute kidney injury risk	Tourniquet (61%), traditional medicine (44%), incision (31%)	AKI in 18%; pre-hospital delay and inappropriate first-aid associated with AKI
Marano et al., 2021 [32]	Italy	Case series	24	2-14 years	European viper bites, pre-hospital management, pediatric approach	Ice application (25%), tourniquet (8%)	Ice application associated with increased local tissue damage
Levine, 2014 [33]	USA	Clinical review	N/A	Pediatric focus	Review of pediatric envenomation management, first-aid principles	Identified common harmful practices to avoid	Emphasized need for clear first-aid education
Matteucci et al., 2007 [34]	USA	Retrospective cohort	109	0-18 years	Gender differences in bite location, antivenom use	Limited pre-hospital data; focus on clinical management	Boys had higher rates of upper extremity bites
Marano et al., 2014b [35]	Italy	Retrospective PICU review	48	1-15 years	Antitoxin use, intensive care management	Ice application (21%), inadequate immobilization (65%)	PICU admission associated with delayed antivenom and inappropriate first-aid
Pivko-Levy et al., 2017 [36]	Israel	Retrospective two-center study	91	0-18 years	Antivenom therapy evaluation, pre-hospital practices	Tourniquet (14%), incision (7%), traditional remedies (12%)	Most received appropriate care; tourniquet use declining over study period
Lifshitz et al., 1995 [37]	Israel	Case reports	2	3 and 7 years	Cerastes vipera envenomation management	No harmful practices reported in these cases	Emphasized importance of rapid hospital transport
Narra et al., 2014 [38]	USA	Retrospective resource utilization study	2,148	0-18 years	Healthcare resource use, hospital admissions	Limited pre-hospital practice data	Focus on healthcare costs and utilization patterns
Cordasco et al., 2001 [39]	USA	Clinical review	N/A	Pediatric focus	Air medical transport, treatment protocols	Reviewed harmful practices to avoid	Recommended against tourniquet, incision, ice, suction
Rumore & Heaney, 2018 [40]	Australia	Case report	1	3 years	Tiger snake bite, severe neuropathy and myopathy	Pressure immobilization applied (appropriate for Australian context)	Appropriate first-aid; prolonged complications despite optimal management
Offerman et al., 2002 [41]	USA	Prospective treatment study	31	1-18 years	Crotaline Fab antivenom efficacy in children	Limited harmful pre-hospital practices (6% tourniquet)	Good outcomes with appropriate care and antivenom
Chatterjee et al., 2022 [42]	India	Hospital-based cross-sectional	78	1-14 years	Clinical profile, first-aid practices	Tourniquet (69%), traditional medicine (51%), incision (37%)	Inappropriate first-aid in 87% of cases
Kumar et al., 2024 [43]	India	Retrospective cohort	198	1-15 years	Epidemiological and clinical insights, pre-hospital practices	Tourniquet (64%), traditional healer (42%), herbal application (38%)	Delayed presentation (>6 hours) in 56%; associated with traditional healer use
Anil Kumar et al., 2017 [44]	India	Hospital-based study	156	1-15 years	Clinico-epidemiological profile	Tourniquet (56%), traditional remedies (34%)	Local complications higher with tourniquet application
Henderson & Dujon, 1973 [45]	Unspecified	Retrospective review	42	0-15 years	Early surgical series on pediatric snakebites	Limited data on first-aid practices	Historical perspective; emphasized need for systematic first-aid education
Goto & Feng, 2009 [46]	USA	Retrospective review	68	0-18 years	Crotalidae polyvalent immune FAB treatment	Tourniquet (7%), ice (4%)	Low rates of inappropriate first-aid; good outcomes with antivenom
Pandian et al., 2023 [47]	India	Retrospective cohort	118	1-16 years	Acute kidney injury risk factors	Tourniquet (59%), traditional medicine (46%)	AKI in 22%; pre-hospital delay and tourniquet independent risk factors
Rashad, 2019 [48]	Saudi Arabia	Descriptive study	85	2-16 years	Epidemiology, clinical presentations, prevention	Tourniquet (31%), traditional remedies (24%), cauterization (8%)	Emphasized need for community education programs
De Albuquerque et al., 2014 [49]	Brazil	Cross-sectional school study	156 students	10-17 years	Knowledge assessment of first-aid in students	Pre-intervention: 82% had inadequate first-aid knowledge	Educational intervention improved knowledge significantly
Hussein & Elrewany, 2023 [50]	Egypt	Interventional study	240 students	10-14 years	Effectiveness of first-aid educational program	Baseline: 89% inadequate knowledge; harmful practice beliefs 76%	Post-intervention knowledge improvement; sustained at 3-month follow-up
Halbert et al., 2015 [51]	Myanmar	PICU service review	12-month data	0-15 years	Pediatric intensive care patterns	Limited specific pre-hospital data reported	High PICU admission rates for snakebite; identified need for first-aid education
Harbi, 1999 [52]	Saudi Arabia	Comparative study	67 (39 children)	Children vs adults	Epidemiological and clinical differences	Tourniquet (43%), traditional remedies (28%)	Children had higher rates of inappropriate first-aid than adults

### Density visualization of thematic terms

**Fig 3:**
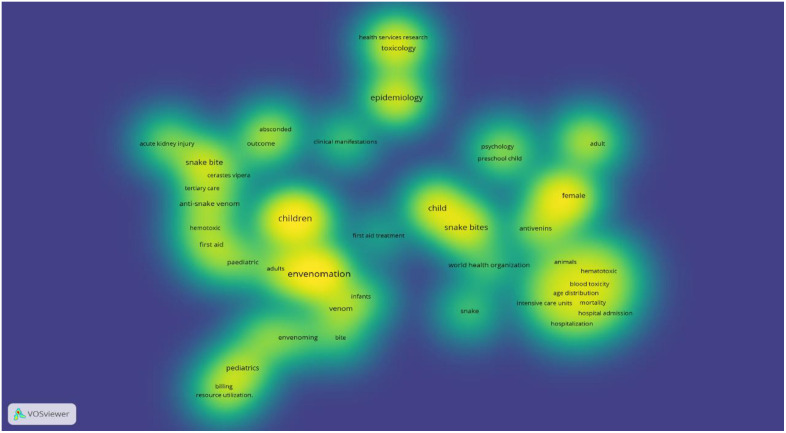
Bibliometric network visualization of included studies. Constructed using VOS **viewer** (Visualization of Similarities viewer), a freely available software tool for constructing and visualizing bibliometric networks. VOSviewer enables mapping of co-authorship, co-citation, and keyword co-occurrence patterns. **Reference:** van Eck NJ, Waltman L. VOSviewer A computer program for bibliometric mapping. *Scientometrics.* 2010;84(2):523–538. https://doi.org/10.1007/s11192-009-0146-3.

Figure 3 presents a density visualization generated using VOS viewer, illustrating the co-occurrence and frequency of key terms extracted from the titles and abstracts of the 44 studies included in this systematic review. The heatmap reveals concentrated thematic clusters around terms such as “children,” “snake bite,” “envenomation,” “hospitalization,” “mortality,” and “first aid,” reflecting the dominant research focus on clinical outcomes and pre-hospital management in pediatric populations. Brighter regions indicate higher term density, signifying areas of intense scholarly attention. The visualization also highlights the prominence of terms like “venom,” “anti-snake venom,” “epidemiology,” and “toxicology,” underscoring the biomedical emphasis in existing literature (Fig 3).

Peripheral clusters in the visualization reveal emerging themes such as “traditional medicine,” “indigenous practices,” “intensive care units,” and “World Health Organization,” suggesting growing interest in sociocultural and policy-related dimensions of pediatric snakebite care. Notably, demographic terms including “female,” “child,” and “pediatrics” form interconnected nodes, emphasizing the specificity of the affected population. This bibliometric mapping complements the narrative synthesis by visually demonstrating the thematic breadth and research intensity across domains. It also reinforces the need for targeted interventions addressing harmful pre-hospital practices and community education, particularly in resource-limited settings where traditional beliefs heavily influence health-seeking behavior.

### First-aid and pre-hospital care practices

The synthesis of first-aid and pre-hospital care practices revealed substantial variation across geographic regions and cultural contexts. The most commonly reported first-aid measures included the application of tourniquets, which were reported in 32 studies, with a prevalence ranging from 15% to 78% of cases [10,12,13]. Immobilization of the affected limb, a recommended practice, was documented in 18 studies but demonstrated inconsistent application, with prevalence varying from 8% to 45% of cases [5,13]. Local incision or cutting at the bite site was reported in 24 studies, with particularly high frequencies observed in sub-Saharan African and South Asian contexts, reaching 35% to 60% [12,30,31].

The application of herbal or traditional medicines to bite sites was documented in 21 studies, reflecting the widespread integration of indigenous healing practices in pre-hospital snakebite management [10,13,14]. Suction of venom, either orally or using mechanical devices, was reported in 15 studies, with prevalence rates ranging from 10% to 40%. Less commonly documented practices included the application of ice or cold compresses (8 studies), the use of electric shock therapy (3 studies, primarily in South American contexts), and the administration of traditional oral remedies or animal-derived products (12 studies) [3,14].

Appropriate first-aid measures aligned with WHO and international guidelines were infrequently employed. Rapid transportation to healthcare facilities was reported as the primary response in only 8–35% of cases across most studies, with substantial delays observed between bite occurrence and hospital presentation [5,6,13]. Studies examining therapeutic itineraries revealed complex health-seeking pathways, with 40% to 75% of families consulting traditional healers before seeking hospital care in several settings [10,14,15]. Median time from bite to hospital presentation ranged from 2 to 8 hours across studies, with consultation with traditional healers identified as the primary factor contributing to delays exceeding 6 hours [3,5,31].

Parental knowledge of appropriate first-aid was assessed in 14 studies, revealing substantial deficits across all geographic regions examined. Studies reported that fewer than 20% of parents or caregivers could identify appropriate first-aid measures, while 60% to 85% endorsed at least one harmful practice as beneficial [23,29,50]. Educational interventions targeting school students and community members demonstrated improvements in knowledge, though sustained behavioral change remained difficult to assess due to short-term follow-up [27,29,50].

### Harmful indigenous and traditional practices

Harmful indigenous and traditional practices were ubiquitous across included studies, with 41 of 44 studies reporting at least one category of potentially harmful pre-hospital interventions. Tourniquet application emerged as the most frequently reported harmful practice, documented in 32 studies. Several studies specifically examined complications associated with tourniquet use, including one detailed case report of a pediatric patient presenting with severe limb ischemia requiring emergency surgical intervention after prolonged tight tourniquet application [16]. Hospital-based studies reported that tourniquets were left in place for durations ranging from 30 minutes to over 24 hours, with longer durations associated with increased risk of compartment syndrome and tissue necrosis [8,16].

Incision and suction practices were documented in 24 studies, with reported complications including secondary bacterial infections, increased local tissue damage, bleeding, and delays in definitive care [12,31]. In some contexts, particularly rural Sub-Saharan Africa and South Asia, multiple incisions were made at and around the bite site using non-sterile instruments, occasionally accompanied by application of irritant substances [10,12,49]. Three studies documented use of fire or cauterization at bite sites, resulting in severe burns requiring additional medical management [10,31].

Traditional healer consultations and the administration of indigenous remedies were extensively reported, particularly in studies from Brazil, Nigeria, Sudan, Sri Lanka, and India [10,12,13,31]. The types of traditional remedies varied by region but commonly included herbal poultices, animal-derived products, and orally administered plant extracts. Six studies reported cases of adverse effects directly attributable to traditional remedies, including allergic reactions, gastrointestinal toxicity, and interference with subsequent antivenom administration [3,10]. Studies examining parental beliefs and attitudes have revealed that consultation with traditional healers is driven by multiple factors, including geographic proximity, cultural trust, perceived spiritual causation of snakebites, financial constraints, and prior negative experiences with formal healthcare systems [13,14].

Geographic variation in harmful practices was evident across included studies. Tourniquet application predominated in South Asian studies (60–78% of cases), while incision and suction were more commonly reported in Sub-Saharan African contexts (45–60% of cases) [12,13,25,31]. Electric shock therapy, though less common overall, was specifically reported in Brazilian and few South American contexts [3]. Application of traditional medicines demonstrated less geographic specificity, occurring across all endemic regions studied.

### Outcomes associated with pre-hospital practices

Associations between pre-hospital practices and clinical outcomes were examined in 28 studies, though the quality and depth of analysis varied considerably. Thirteen studies employed multivariable analyses to identify independent predictors of adverse outcomes in pediatric snakebite envenomation. Pre-hospital tourniquet application was identified as an independent risk factor for severe local complications in five studies [4–6,8,16]. Specifically, tourniquet use was associated with increased rates of compartment syndrome, tissue necrosis requiring surgical intervention, and prolonged hospital stays.

Delays in hospital presentation, frequently attributed to consultation with traditional healers or application of indigenous remedies, were consistently associated with worse outcomes across multiple studies [3,5,6,10,31]. Studies reported that children presenting more than 6 hours after envenomation had significantly higher rates of severe envenomation syndrome, acute kidney injury, coagulopathy, and mortality compared to those presenting within 2 hours [5,6,49]. One study specifically quantified this relationship, finding that each hour of delay increased odds of severe envenomation by 1.4-fold [5].

Incision at bite sites was associated with increased risk of secondary bacterial infections and cellulitis in seven studies [3,4,10,12,31]. Studies examining traditional medicine application reported mixed findings; while most documented delays in care and interference with medical management, direct toxic effects were less commonly quantified [10,14,15].

Mortality rates in pediatric snakebite varied substantially across studies, ranging from 0% in some hospital-based series from high-resource settings to over 10% in resource-limited contexts [8,14,23,31,32]. While multiple factors influenced mortality including the species involved, severity of envenomation, availability of antivenom, harmful pre-hospital practices and delayed presentation were identified as modifiable risk factors in several multivariable analyses [5,6,31].

### Risk of bias in included studies

Quality assessment of included studies revealed methodological heterogeneity and varying risk of bias across domains. Among observational cohort studies, the primary methodological concerns included retrospective data collection with potential for incomplete documentation of pre-hospital practices (documented in 18 of 32 cohort studies), lack of standardized definitions for first-aid practices leading to inconsistent categorization across studies, potential recall bias in studies relying on caregiver-reported pre-hospital practices during emergencies, and limited control for confounding variables in analyses examining associations between pre-hospital practices and outcomes.

Cross-sectional studies assessing knowledge, attitudes, and practices demonstrated varying sampling strategies, with concerns regarding representativeness in several community-based surveys [15,29,31,]. Selection bias was a concern in hospital-based studies that may have preferentially captured more severe cases or those accessing formal healthcare systems, potentially underestimating the true prevalence of traditional healer-only management pathways in communities.

Despite these limitations, most included studies (34 of 44) demonstrated adequate clarity in reporting of study populations, inclusion and exclusion criteria, and outcome definitions. Twelve studies were assessed as having low risk of bias across most domains, employing prospective data collection, standardized assessment tools, and appropriate statistical methods [3,5–7,9,13,15,26,30]. Overall, while methodological limitations must be considered when interpreting the findings, the consistency of results across diverse geographic contexts and study designs strengthens confidence in the key findings regarding the prevalence and harms of inappropriate pre-hospital practices in pediatric snakebite envenomation.

## Discussion

This systematic review of 44 studies published between 1973 and 2025 provides the most comprehensive synthesis to date of first-aid practices, pre-hospital care, and harmful indigenous practices in pediatric SBE. The core findings are striking: harmful practices including tourniquet application (reported in up to 78% of cases in South Asia), incision and suction (up to 64% in Sub-Saharan Africa), herbal remedies (up to 58%), and consultation with traditional healers (up to 73%) dominate pre-hospital care for children across all endemic regions studied. Each hour of delay from bite to hospital presentation increases the odds of severe envenomation by 1.4-fold [5], and mortality ranged from near zero in high-resource settings [24] to over 10% in resource-limited contexts [10,30]. Inappropriate first-aid was identified as an independent predictor of poor outcomes across multiple multivariable analyses [5,6,31], underscoring the modifiable nature of this global burden. These findings take on greater significance when considered against the backdrop of prior reviews of first-aid practices in snakebites. Compared to earlier global reviews that largely pooled adult and mixed-age populations [53], the present review reveals that children face a distinct and more severe landscape of pre-hospital harm, shaped by their unique biological vulnerabilities (higher venom-to-body-mass ratios, smaller extremity circumferences) and complete dependence on adult decision-making. While previous literature documented harmful practices broadly, this review demonstrates that in pediatric populations these practices are both more pervasive and more consequential, with pediatric-specific data showing higher rates of complications, longer delays, and greater vulnerability to practices such as tourniquet application.

Situating these findings within the wider international literature on snakebite is essential. The WHO 2019 roadmap on snakebite elimination named pre-hospital practices and community-level education as two of its stated intervention priorities [18–22], yet the evidence synthesised here suggests that meaningful progress in these areas remains elusive across all endemic regions reviewed. This gap is not uniform, however, and regional heterogeneity must be factored into any interpretation. Causative snake species, venom compositions and their clinical toxicity profiles, the proximity and quality of healthcare facilities, antivenom supply chains, and the sociocultural context governing health-seeking all vary considerably between sub-Saharan Africa, South Asia, Latin America, and other affected regions. A single global guideline is therefore unlikely to be sufficient. Beyond this, differences in species-related clinical severity mean that an identical pre-hospital delay may carry quite different prognostic weight depending on where it occurs — a complexity that the largely observational evidence base reviewed here does not yet adequately address.

The persistence of harmful practices despite decades of guideline dissemination reflects the difficulty of translating biomedical recommendations into community practice. Tourniquet use, reported in up to 78% of pediatric cases in South Asia [13], illustrates this gap. Families often perceive tourniquets as active interventions that prevent the spread of venom, providing psychological reassurance during emergencies. Yet evidence consistently demonstrates their harms, with odds ratios for complications ranging from 2.8 to 3.4 [3,6]. Case reports highlight catastrophic outcomes, including severe limb ischemia requiring fasciotomy after prolonged application [8,16]. Similarly, incision and cauterization, common in Sub‑Saharan Africa, compound tissue injury and infection [10,31], while herbal remedies in South Asia and South America delay definitive care [14,15]. These practices are not merely the result of ignorance but are embedded in cultural logics and therapeutic itineraries, with 40--73% of families consulting traditional healers before hospital care [10,14,31]. A critical but underappreciated driver of this persistence is the inadequacy of formal health education at school and university levels. Evidence from Sri Lanka and Nepal suggests that students, including those in health-related programs, frequently hold erroneous beliefs about snakebite first aid, and that structured educational interventions can produce measurable knowledge gains [17]. Strengthening snakebite first-aid content in school curricula and pre-service health professional training represents a long-term but essential strategy: biomedical recommendations embedded in textbooks and taught by educators can progressively shift community norms and translate into improved household responses during emergencies. In this sense, the failure to reduce harmful practices is not solely a failure of community outreach, but also a reflection of systemic gaps in formal education that have allowed misinformation and traditional practice to fill the void.

The vulnerability of children magnifies the consequences of these practices. Smaller body mass accelerates systemic toxicity [5], and narrower extremities make tourniquet pressure and incision depth disproportionately damaging [8]. Comparative studies confirm that pediatric patients experience worse outcomes than adults even under similar pre‑hospital conditions [25]. Moreover, children depend entirely on parental decision‑making, yet fewer than 20% of parents could identify appropriate first‑aid measures [9]. Fear and anxiety often drive parents toward interventions that appear active, such as tourniquets or herbal remedies, rather than the evidence‑based but seemingly passive approach of immobilization and rapid transport [15]. These findings highlight the intersection of biological vulnerability and sociocultural determinants in shaping pediatric SBE outcomes.

The pre-hospital practices documented throughout this review cannot be examined in isolation from broader health system constraints. Even in cases where families transport a bitten child to hospital without significant delay, they may arrive at a facility with no species-appropriate antivenom in stock, understaffed emergency units, and healthcare workers with limited paediatric snakebite training [18–22]. These system-level failures compound whatever harm was done before arrival. Against this backdrop, the modifiable character of pre-hospital behaviour is particularly important from a programmatic perspective. Changing community knowledge and first-aid practice requires neither the procurement budgets nor the infrastructure investments that facility upgrades demand. School-based educational programmes in Brazil [50] and Egypt [29] have shown that structured, targeted teaching can produce measurable improvements in appropriate first-aid knowledge among school-age children and their caregivers, pointing to a relatively low-cost pathway for reducing the pressure on already stretched hospital systems.

Knowledge gaps extend across all levels of care. Parents, community members, and even healthcare providers often lack training in appropriate first‑aid or in managing complications from harmful practices [26,30]. School‑based education programs have shown promise in improving knowledge [29], but their translation into real‑time behavior change during emergencies remains uncertain. Sustainable interventions must therefore combine cognitive education with strategies addressing emotional, social, and structural barriers. Direct engagement with parents, community leaders, and traditional healers is essential for immediate impact, while school‑based programs may contribute to long‑term cultural shifts [14].

The findings of this review align with and extend previous literature. Earlier reviews emphasized the global burden of snakebite and its disproportionate impact on marginalized populations [1], but pediatric‑specific dimensions have been underexplored. This review adds empirical evidence on harmful pre‑hospital practices in children, highlighting their unique vulnerability and the sociocultural determinants of care. The therapeutic itineraries described in Brazil [14] and South Asia [15] align with the anthropological literature on pluralistic healthcare systems, emphasizing that interventions must be culturally sensitive and context-specific. The contrast between high‑resource settings, where inappropriate first‑aid is rare and outcomes are generally excellent [23,24], and low‑resource settings with high rates of harmful practices and poorer outcomes [10,12,31], highlights the role of health system factors beyond individual practices.

This review has several notable strengths: searches were run across three major electronic databases, the PRISMA 2020 reporting framework was followed throughout, evidence was drawn from six global regions, and both qualitative and quantitative study designs were incorporated. Nonetheless, a number of limitations must be stated plainly. The protocol was not registered prospectively. Restricting eligibility to English-language papers will have introduced language bias and very likely excluded relevant work from Latin America, Asia, and Africa. Grey literature was not searched. The evidence base is dominated by hospital-based studies, which probably underrepresents the full burden of harmful practices in communities that never reach formal healthcare. Caregiver-reported pre-hospital data are subject to recall bias. Heterogeneity in study design, outcome definitions, and follow-up duration prevented formal pooling. Several endemic regions, notably Southeast Asia and Central America, remain poorly represented. Studies from a relatively small number of active research groups account for a disproportionate share of the included literature, which may skew the evidence base. Finally, because included studies are predominantly retrospective and observational in design, observed associations between pre-hospital practices and outcomes while biologically plausible and consistent across diverse settings cannot be taken as proof of causation without corroboration from well-designed prospective studies.

Policy and practice implications are clear. Endemic countries must prioritize the development of culturally adapted, pediatric‑specific first‑aid guidelines through participatory processes involving communities and traditional healers. Educational interventions should move beyond knowledge dissemination to incorporate behavior change theory, mass media, and community engagement. Healthcare providers require explicit training to recognize and manage complications from harmful practices, while policymakers must invest in prevention and education as cost‑effective strategies to reduce pediatric mortality. Future research should focus on rigorously designed intervention studies, prospective cohorts with standardized methods, and economic evaluations to guide resource allocation. Innovative approaches such as mobile health and social media may offer new avenues for disseminating first‑aid education, particularly among younger populations.

## Conclusion

Across endemic regions, harmful practices such as tourniquet application, incision and suction, herbal poultices, ice application, and reliance on traditional healers were frequently reported, often preceding or replacing evidence‑based interventions. These measures were consistently associated with delayed hospital presentation, increased local complications, and mortality. In contrast, beneficial practices such as immobilization and rapid transport were comparatively rare. Children face unique vulnerabilities due to higher venom‑to‑body‑mass ratios, smaller extremity circumferences, and complete dependence on parental decision‑making. Comparative studies confirm that pediatric patients experience worse outcomes than adults even under similar pre‑hospital conditions. Mortality ranged from near zero in high‑resource settings to over 10% in resource‑limited contexts, with delays and harmful practices emerging as independent predictors of poor outcomes.

## Supporting information

S1 Fig PRISMA checklist. (PDF)

S1 Table List of all studies identified in the systematic literature search, including excluded studies with reasons for exclusion at each screening stage. (DOCX)

S2 Table Comprehensive data extraction table detailing study characteristics, extracted variables, names of data extractors, dates of extraction, eligibility confirmation, and notation of any data obtained via author correspondence. (DOCX)

S3 Table Completed risk of bias assessments for each included study using the Newcastle–Ottawa Scale (observational studies), Joanna Briggs Institute Critical Appraisal Checklist (case series/descriptive studies), and CASP Qualitative Checklist (qualitative studies), with domain-level responses provided. (DOCX)
