# Supplementary material for: First-aid, pre-hospital care, and harmful indigenous practices in pediatric snakebite envenomation: A systematic review of global evidence from 1973 to 2025
Source: PLoS Negl Trop Dis. 2026 Jul 28;20(7):e0014508. doi: 10.1371/journal.pntd.0014508 (PMC13411870; doi:10.1371/journal.pntd.0014508)
Supplement: S1 Table — (DOCX) [file pntd.0014508.s002.docx]

**S1 Table:** List of all studies identified in the systematic literature search, including excluded studies with reasons for exclusion at each screening stage

Total records identified from databases: 655 (Scopus: 270; PubMed: 294; Lens.org: 91). After removing 193 duplicates, 462 records were screened. Following title/abstract screening (367 excluded) and full-text review (42 excluded from 86 assessed; 9 not retrieved), 44 studies met all inclusion criteria and are included in the systematic review. Representative records excluded at title/abstract screening are shown below. All 42 studies excluded at full-text review are listed with reasons.

| **No.** | **Author(s), Year** | **Title** | **DOI** | **Country** | **Study Design** | **Decision** |
| --- | --- | --- | --- | --- | --- | --- |
| 1 | Cristino et al., 2025 | Therapeutic itineraries of children after snakebites in the Brazilian Amazon | 10.1371/journal.pntd.0013777 | Brazil | Qualitative thematic study | **Included** |
| 2 | Dayasiri et al., 2025a | First-aid practices and pre-hospital care in paediatric snakebites | 10.1186/s12887-025-05975-0 | Sri Lanka | Cross-sectional survey | **Included** |
| 3 | Dayasiri et al., 2025b | Training and educational needs for paediatric snakebite management among rural healthcare providers in Sri Lanka | 10.1016/j.toxicon.2025.108459 | Sri Lanka | Cross-sectional survey of HCPs | **Included** |
| 4 | Dayasiri et al., 2025c | Challenges in paediatric snakebite management: physician perspectives from rural Sri Lanka | 10.1016/j.toxicon.2025.108410 | Sri Lanka | Qualitative interviews | **Included** |
| 5 | Dayasiri et al., 2025d | Preventive practices and parental attitudes towards snakebites in children in snakebite hotspots of rural Sri Lanka | 10.1136/bmjpo-2025-003543 | Sri Lanka | Cross-sectional household survey | **Included** |
| 6 | Dayasiri et al., 2025e | Caught between fear and tradition: parental knowledge, beliefs and emergency responses to paediatric snakebites in rural Sri Lanka | 10.1136/bmjpo-2025-003658 | Sri Lanka | Mixed-methods study | **Included** |
| 7 | Oliveira et al., 2023 | Snakebite envenoming in Brazilian children: clinical aspects, management and outcomes | 10.1093/tropej/fmad010 | Brazil | Retrospective cohort | **Included** |
| 8 | Buitendag et al., 2021b | A comparison between adult and paediatric snakebites and their outcomes in North Eastern South Africa | 10.1016/j.toxicon.2021.12.009 | South Africa | Comparative cohort | **Included** |
| 9 | Suryanarayana et al., 2020 | Retrospective hospital-based cohort study on risk factors of poor outcome in pediatric snake envenomation | 10.1093/tropej/fmaa078 | India | Retrospective cohort | **Included** |
| 10 | Geyt et al., 2020 | Paediatric snakebite envenoming: recognition and management of cases | 10.1136/archdischild-2020-319428 | Multi-country | Narrative review with cases | **Included** |
| 11 | Nduagubam et al., 2020 | Snakebite in children in Nigeria: A comparison of first aid treatment measures with WHO guidelines | 10.4103/aam.aam_38_19 | Nigeria | Cross-sectional hospital-based | **Included** |
| 12 | Variawa et al., 2020 | Prospective review of cytotoxic snakebite envenomation in a paediatric population | 10.1016/j.toxicon.2020.12.009 | South Africa | Prospective review | **Included** |
| 13 | Giri et al., 2020 | Snake bite, a neglected menace: a prospective observational study in a tertiary care PICU | 10.18203/2349-3291.ijcp20201646 | India | Prospective observational | **Included** |
| 14 | Sood et al., 2020 | Epidemiological and clinical profile of paediatric snake bite patients at a tertiary care centre of Himachal Pradesh, India | 10.18203/2349-3291.ijcp20202143 | India | Retrospective cohort | **Included** |
| 15 | Pandey et al., 2020 | School students' perceptions on snakes, their uses, and snakebite in Nepal | 10.23880/act-16000180 | Nepal | Cross-sectional school survey | **Included** |
| 16 | Bush & Kinlaw, 2015 | Management of a pediatric snake envenomation after presentation with a tight tourniquet | 10.1016/j.wem.2015.01.005 | USA | Case report with management review | **Included** |
| 17 | Sankar et al., 2013 | Factors affecting outcome in children with snake envenomation: a prospective observational study | 10.1136/archdischild-2012-303025 | India | Prospective observational | **Included** |
| 18 | Mars et al., 1991 | Direct intracompartmental pressure measurement in the management of snakebites in children | N/A (PubMed) | South Africa | Case series | **Included** |
| 19 | Tadros et al., 2022 | Emergency department visits by pediatric patients for snakebites | 10.1097/PEC.0000000000002725 | USA | Retrospective ED database analysis | **Included** |
| 20 | Schulte et al., 2016 | Childhood victims of snakebites: 2000-2013 | 10.1542/peds.2016-0491 | USA | Retrospective national database | **Included** |
| 21 | Sanni et al., 2021 | Prevalence and outcome of snake bites among children admitted in the Emergency Pediatric Unit, Federal Medical Centre, Birnin Kebbi, Nigeria | 10.7759/cureus.17413 | Nigeria | Prospective cohort | **Included** |
| 22 | Pattanaik et al., 2023 | Clinical, laboratory profile and outcomes in children with snakebite from Eastern India | 10.4103/jfmpc.jfmpc_1965_22 | India | Retrospective cohort | **Included** |
| 23 | Ahmed et al., 2019 | Snake bites envenoming among children in Gadarif, Eastern Sudan | N/A | Sudan | Descriptive cross-sectional | **Included** |
| 24 | Marano et al., 2021 | Acute exposure to European viper bite in children: advocating for a pediatric approach | 10.3390/toxins13050330 | Italy | Case series | **Included** |
| 25 | Levine, 2014 | Pediatric envenomations: don't get bitten by an unclear plan of care | N/A (PubMed) | USA | Clinical review | **Included** |
| 26 | Matteucci et al., 2007 | Pediatric sex group differences in location of snakebite injuries requiring antivenom therapy | 10.1007/bf03160919 | USA | Retrospective cohort | **Included** |
| 27 | Marano et al., 2014b | Antitoxin use and pediatric intensive care for viper bites in Rome, Italy | N/A (PubMed) | Italy | Retrospective PICU review | **Included** |
| 28 | Pivko-Levy et al., 2017 | Evaluation of antivenom therapy for Vipera palaestinae bites in children | 10.1080/15563650.2016.1277233 | Israel | Retrospective two-center study | **Included** |
| 29 | Lifshitz et al., 1995 | Snake bite by Cerastes vipera in children: report of two cases | 10.1580/1080-6032(1995)006 | Israel | Case reports | **Included** |
| 30 | Narra et al., 2014 | Resource utilization of pediatric patients exposed to venom | 10.1542/hpeds.2014-0010 | USA | Retrospective resource utilization | **Included** |
| 31 | Cordasco et al., 2001 | Treatment of the pediatric snakebite victim | 10.1016/s1067-991x(01)70091-0 | USA | Clinical review | **Included** |
| 32 | Rumore & Heaney, 2018 | Severe protracted neuropathy and myopathy in a 3-year-old girl following tiger snake bite | 10.1111/jpc.14066 | Australia | Case report | **Included** |
| 33 | Offerman et al., 2002 | Crotaline Fab antivenom for the treatment of children with rattlesnake envenomation | 10.1542/peds.110.5.968 | USA | Prospective treatment study | **Included** |
| 34 | Chatterjee et al., 2022 | Snake bite in children – a hospital-based cross-sectional study in a tertiary care hospital in Eastern India | 10.36106/ijar/0702232 | India | Hospital-based cross-sectional | **Included** |
| 35 | Kumar et al., 2024 | Understanding pediatric snakebites: clinical and epidemiological insights from a healthcare center in Bihar, India | 10.4103/jfmpc.jfmpc_1817_23 | India | Retrospective cohort | **Included** |
| 36 | Anil Kumar et al., 2017 | Clinico-epidemiological profile of snake bite in children in a tertiary care centre | 10.18203/2349-3291.ijcp20175572 | India | Hospital-based study | **Included** |
| 37 | Henderson & Dujon, 1973 | Snake bites in children | 10.1016/0022-3468(73)90414-4 | Not specified | Retrospective review | **Included** |
| 38 | Goto & Feng, 2009 | Crotalidae polyvalent immune FAB for the treatment of pediatric crotaline envenomation | 10.1097/pec.0b013e31819f1f1e | USA | Retrospective review | **Included** |
| 39 | Pandian et al., 2023 | Clinic-epidemiological profile and acute kidney injury among pediatric snake bite cases | 10.5455/njppp.2023.13.02094202319022023 | India | Retrospective cohort | **Included** |
| 40 | Rashad, 2019 | Snakebites in Al-Baha District, Saudi Arabia: epidemiology, clinical presentations, management and prevention | 10.1136/archdischild-2019-epa.620 | Saudi Arabia | Descriptive study | **Included** |
| 41 | De Albuquerque et al., 2014 | Saving lives: assessing knowledge of students of a public school about first aid | 10.5205/6656 | Brazil | Cross-sectional school study | **Included** |
| 42 | Hussein & Elrewany, 2023 | Effectiveness of first aid educational program on the knowledge of primary school students in Egypt | 10.21608/jhiph.2023.332645 | Egypt | Interventional study | **Included** |
| 43 | Halbert et al., 2015 | A twelve month review of paediatric intensive care in Myanmar to guide service development | 10.1136/archdischild-2015-308599.251 | Myanmar | PICU service review | **Included** |
| 44 | Harbi, 1999 | Epidemiological and clinical differences of snake bites among children and adults in south western Saudi Arabia | 10.1136/emj.16.6.428 | Saudi Arabia | Comparative study | **Included** |

| **EXCLUDED AT TITLE/ABSTRACT SCREENING** | | | |
| --- | --- | --- | --- |
| **No.** | **Title** | **DOI** | **Reason for rejection** |
| E1 | Pre-hospital interventions in snakebite: A telephonic survey from Coastal Karnataka | 10.1371/journal.pntd.0013334 | Not pediatric-specific / adult mixed population |
| E2 | Predictors and nomogram for amputation risk in pit viper snakebite envenoming at hospital admission | 10.1038/s41598-025-26903-3 | Not pediatric-specific; adult population; no first-aid component |
| E3 | Case Series of Snakebites and Complicated Pit Viper Envenomation Management in Central America | 10.4269/ajtmh.24-0720 | Not pediatric-specific; no pre-hospital/first-aid data |
| E4 | The impact of the COVID-19 pandemic on snakebite patterns in rural Sri Lanka | 10.1016/j.toxicon.2025.108502 | No first-aid/pre-hospital component; epidemiology only |
| E5 | The clinical significance and management of finger necrosis due to cobra bite in Vietnam | 10.1016/j.jpra.2025.05.011 | Not pediatric; in-hospital management only |
| E6 | Characteristics of snakebite patients due to Naja samarensis in the Philippines | 10.1093/trstmh/trae110 | Not pediatric-specific; no pre-hospital data |
| E7 | Health literacy among rural Bangladeshi population on first aid and prevention of snakebite | 10.1093/trstmh/trae130 | Not pediatric population; adult community study |
| E8 | Clinico-epidemiological study of snakebite: 13 years of data from eastern Nepal | 10.1093/trstmh/trae119 | Mixed adult/child population; no pediatric-specific data |
| E9 | Self-reported health effects after one year from a viper bite in France | 10.1016/j.toxicon.2025.108360 | Not pediatric; adult cohort; no first-aid data |
| E10 | Symptoms and Management of Painful Progressive Swelling in Eswatini Snakebite Patients | 10.4269/ajtmh.24-0671 | Not pediatric; in-hospital management; no pre-hospital data |
| E11 | Assessing Epidemiological and Demographic Features of Snakebite in Khuzestan Province | 10.5812/jjnpp-161011 | Not pediatric; descriptive epidemiology only |
| E12 | Determinants of seeking allopathic treatment after snakebite in rural Sri Lanka | 10.1093/trstmh/trae071 | Adult population; health-seeking in general, not pediatric-specific |
| E13 | Survey on snakebite burden in the HDSS of Taabo, Southern Côte d'Ivoire | 10.1371/journal.pntd.0012983 | Not pediatric-specific; general epidemiology |
| E14 | Health-seeking behaviours and traditional healer practices for snakebite in southern India | 10.1093/trstmh/trae083 | Adult population; no pediatric-specific data |
| E15 | Extremity fasciotomy in the developing world – a South African experience | 10.36303/SAJS.00675 | Surgical management only; not pediatric-specific; no first-aid data |
| E16 | A Clinico-Epidemiological Study on Green Pit Viper Bite in Chittagong Medical College Hospital | 10.3329/jom.v26i1.78993 | Not pediatric-specific; mixed adult/child; in-hospital data |
| E17 | Participatory research towards control of snakebite in the Western Brazilian Amazon | 10.1371/journal.pntd.0012840 | Not pediatric-specific; community general population |
| E18 | 2024 American Heart Association and American Red Cross Guidelines for First Aid | 10.1161/CIR.0000000000001281 | Clinical guideline/review — no original pediatric snakebite data |
| E19 | A supervised machine learning approach to modeling barriers to snakebite treatment in Ghana | 10.1371/journal.pntd.0012736 | Not pediatric; adult model; no first-aid component |
| E20 | Faith healing: the threat of 'Surucucu' and local cure of Amazon floodplain dwellers | 10.1186/s13002-024-00715-7 | Not pediatric-specific; ethnobotanical/general population study |
| E21 | Vulnerability factors of snake bite patients in China | 10.1186/s12889-024-19169-3 | Not pediatric-specific; adult population; epidemiology only |
| E22 | Factors associated with complications of snakebite envenomation in Burkina Faso | 10.3390/tropicalmed9110268 | Not pediatric; general population; in-hospital management |
| E23 | Snakebite incidence and healthcare-seeking in Eastern Province, Rwanda | 10.1371/journal.pntd.0012378 | Not pediatric-specific; general population study |
| E24 | Therapeutic itineraries of snakebite victims and antivenom access in southern Mexico | 10.1371/journal.pntd.0012301 | Not pediatric-specific; adult population |
| E25 | Venomous snakebites in children: a 10 year experience in South China | 10.1080/15563650.2024.2341124 | In-hospital management only; no pre-hospital/first-aid data |
| E26 | Clinico-Epidemiological Profile of snakebite in Eastern India (1-year prospective) | 10.1177/10806032241239628 | Not pediatric-specific; mixed adult population |
| E27 | Domestic Accidents of Children in Orodara District, Burkina Faso: Mothers' First Aid | 10.3390/ijerph21050523 | Not snakebite-specific; general first-aid study |
| E28 | Prevention & management of snakebite in Maharashtra & Odisha: qualitative study | 10.25259/ijmr_1566_23 | Not pediatric-specific; adult/general community |
| E29 | A randomized controlled trial investigating antivenom for red-bellied black snake envenomation | 10.1080/15563650.2024.2367677 | Not pediatric; clinical trial in adults; in-hospital only |
| E30 | Baseline Knowledge and Change in Confidence after First Aid Training among High School Red Cross Cadets | 10.4103/ijph.ijph_1458_22 | General first aid program, not pediatric snakebite-specific outcomes |
| E31 | Clinical Characteristics and Management of Snake Bite in Jerusalem Area | 10.3390/jcm12124132 | Not pediatric-specific; mixed adult/child; in-hospital focus |
| E32 | Compartment syndrome following snakebite in a boy: case report and literature review | 10.1016/j.ijscr.2023.108050 | Single case report — excluded by design |
| E33 | Risks of snakebite and challenges to seeking treatment in Tanzania agro-pastoral communities | 10.1371/journal.pone.0280836 | Not pediatric-specific; adult general population |
| E34 | Assessment of snakebite management practices at Meserani Juu, Northern Tanzania | 10.1371/journal.pone.0278940 | Not pediatric-specific; general population |
| E35 | Nonvenomous Snakebite in Pediatric Age Group | 10.1055/s-0042-1750376 | Non-venomous bites only; no envenomation/first-aid data |
| E36 | Clinical profile of snake bite in children at a tertiary care center | 10.37506/ijfmt.v14i4.11514 | Insufficient methodological detail; duplicate demographic data |
| E37 | Pattern of snake bite cases at tertiary health care centre and first aid treatment | Not available | Insufficient data quality/unclear methodology; no DOI |
| E38 | A Retrospective descriptive study of snake bites in Alipurduar hospital, India | Not available | Insufficient data quality; no DOI; duplicate cohort concern |
| E39 | Severity grading, management and outcome of snakebite in South India: 5 years | 10.37506/v14/i1/2020/ijfmt/192872 | Not pediatric-specific; adult population; in-hospital only |
| E40 | Epidemiological and clinical aspects of snakebites in the Upper Juruá River Region, Brazil | 10.1590/1809-4392201901561 | Not pediatric-specific; adult predominant; no pediatric sub-analysis |
| E41 | Inadequate knowledge about snakebite envenoming in Myanmar community | 10.1371/journal.pntd.0007171 | Not pediatric-specific; adult community survey |
| E42 | Management and cost of snakebite at a teaching hospital in western Kenya | 10.12688/f1000research.20268.1 | Not pediatric-specific; mixed adult population |
| E43 | Why snakebite patients in Myanmar seek traditional healers | 10.1371/journal.pntd.0006299 | Not pediatric-specific; adult qualitative study |
| E44 | Pre-hospital care and its association with clinical outcome in South India | 10.1177/0049475520966958 | Not pediatric-specific; adult cohort |
| E45 | Compartment syndrome following snakebite (case report) – France | Not available | Single case report — excluded by design |
| E46 | Snake envenomation and pre-hospital care in Nigeria: delay study | Not available | No DOI; insufficient data; adult-focused |
| E47 | Paediatric emergencies and related mortality in Nicaragua | 10.1136/emermed-2019-209324 | Snakebite not primary focus; no snakebite-specific pre-hospital data |
| E48 | Systematic review of human poisoning in Myanmar | 10.3390/ijerph18073576 | Systematic review/secondary analysis; no original pediatric snakebite data |
| E49 | Clinical profile of snake bite in children (Bangalore tertiary care) | Not available | Duplicate cohort with another included study |
| E50 | Impact of first aid training in management of snake bite victims in Madi valley | Not available | No DOI; insufficient methodological detail for inclusion |
| E51 | Venomous snakebites in two children | Not available | Case reports (n=2); insufficient data; no DOI |
| E52 | Amputation of a limb secondary to snakebite in a child | 10.1016/j.arcped.2016.12.013 | Single case report — excluded by design |
| E53 | A retrospective review of rattlesnake bites in 100 children | 10.23736/S0026-4946.16.04226-2 | In-hospital focus only; minimal pre-hospital data; duplicate concern |
| E54 | Myocarditis complicating viper snake bite in a child | 10.22506/ti/2017/v24/i2/162427 | Single case report — excluded by design |
| E55 | Snake bite by Cerastes vipera in children: report of two cases (see Lifshitz) | 10.1580/1080-6032(1995)006 | Duplicate — same paper as Lifshitz 1995; already captured |
| E56 | Hyperglycemia as a risk factor in children after European viper bites | 10.3109/15563650.2015.1113542 | Not pre-hospital focus; in-hospital clinical study |
| E57 | Venomous snakebites in children in southern Croatia | 10.1016/j.toxicon.2016.01.057 | In-hospital management only; no pre-hospital data |
| E58 | Risk factors for high-grade envenomations after French viper bites in children | 10.1097/PEC.0b013e31825cfd66 | In-hospital risk factor study; no pre-hospital data |
| E59 | Coral snake bites and envenomation in children: A case series | 10.1097/PEC.0000000000000109 | Case series without pre-hospital data; insufficient methodology |
| E60 | Transporting children with toxicological emergencies | 10.1111/1742-6723.12221 | Not snakebite-specific; general toxicology transport review |
| E61 | Controversies in treatment of pediatric crotalinae snake envenomation | 10.1016/S1522-8401(01)90005-9 | Review/guideline; no original pre-hospital data |
| E62 | Suspected snakebite in children: A study of 156 patients over 10 years | 10.5694/j.1326-5377.1996.tb122122.x | In-hospital retrospective; limited pre-hospital data; methodology unclear |
| E63 | Diagnosis and treatment of confirmed and suspected snake bite: 46 paediatric cases | 10.5694/j.1326-5377.1992.tb139749.x | In-hospital management focus; no first-aid data |
| E64 | Crotalidae envenomation in children | 10.1097/00000637-199308000-00009 | In-hospital management only; no pre-hospital data |
| E65 | An epidemiological and clinical study of snake-bites in childhood (Australia 1989) | 10.5694/j.1326-5377.1989.tb136764.x | Primarily in-hospital; insufficient pre-hospital data; very old |
| E66 | Envenomation by the Northern Blacktail rattlesnake: case report | 10.1097/01.pec.0000150989.03981.06 | Single case report — excluded by design |
| E67 | Snakebite: A Pediatric Problem (1965 editorial) | 10.1177/000992286500400413 | Editorial/opinion — excluded by design; no original data |
| E68 | Controversies and hazards in treatment of pit viper bites | 10.1097/00007611-197908000-00002 | Old review/opinion; no original pediatric pre-hospital data |
| E69 | Snake Bite in Children: A Five Year Study from South-East Queensland | 10.1111/j.1440-1754.1978.tb02993.x | In-hospital management; limited/no pre-hospital data |
| E70 | Venomous snakebite in Florida (historical) | Not available | No DOI; editorial/historical note; no original data |
| E71 | Snakebite admissions in Zimbabwe: pattern, clinical presentation and management | Not available | Not pediatric-specific; adult hospital data; no DOI |
| E72 | Saving lives: assessing knowledge of school students — duplicate check | 10.5205/6656 | Included (see study 41) — this entry is a duplicate search hit |
| E73 | Pre-hospital management and outcome of acute poisonings by ambulances in Russia | 10.3109/15563650.2013.827707 | Not snakebite-specific; general poisoning; not pediatric |
| E74 | Clinical features and treatment of 292 Chinese cobra snakebites | 10.1016/j.etap.2013.12.018 | Not pediatric-specific; adult population; in-hospital focus |
| E75 | The effect of pre-hospital care for venomous snake bite on outcome in Nigeria | 10.1016/j.trstmh.2010.09.005 | Not pediatric-specific; adult cohort |
| FT1 | Venomous snakebites in children in South China (full text) | 10.1080/15563650.2024.2341124 | In-hospital management only; no pre-hospital/first-aid outcomes data on pediatric subgroup |
| FT2 | Clinical profile of snake bite in children at a tertiary care center | 10.37506/ijfmt.v14i4.11514 | Insufficient data quality; inadequate methodology description; duplicate cohort concern |
| FT3 | Pattern of snake bite cases at tertiary health care centre and first aid treatment | Not available | No accessible full text; insufficient methodological detail; no DOI |
| FT4 | Retrospective descriptive study of snake bites at Alipurduar hospital, India | Not available | Unable to retrieve full text (no DOI); insufficient data quality |
| FT5 | Severity grading and outcomes of snakebite in South India (5 years retrospective) | 10.37506/v14/i1/2020/ijfmt/192872 | Not pediatric-specific; adult cohort; no pediatric subgroup data |
| FT6 | Epidemiological and clinical aspects of snakebites in Upper Juruá, Western Brazilian Amazonia | 10.1590/1809-4392201901561 | Mixed adult/child population; no separate pediatric pre-hospital analysis |
| FT7 | Inadequate knowledge about snakebite envenoming in Myanmar community | 10.1371/journal.pntd.0007171 | Adult community-based study; no pediatric-specific pre-hospital outcomes data |
| FT8 | Management and cost of snakebite injuries at a teaching hospital in western Kenya | 10.12688/f1000research.20268.1 | Not pediatric-specific; mixed population; in-hospital costs focus |
| FT9 | Why snakebite patients in Myanmar seek traditional healers despite hospital availability | 10.1371/journal.pntd.0006299 | Adult qualitative study; no pediatric-specific data |
| FT10 | Pre-hospital care and clinical outcome of snakebite in South India | 10.1177/0049475520966958 | Adult cohort only; no pediatric subgroup; in-hospital management focus |
| FT11 | Compartment syndrome following snakebite in a boy (case report) | 10.1016/j.ijscr.2023.108050 | Single case report excluded by design |
| FT12 | Paediatric emergencies and related mortality in Nicaragua (registry study) | 10.1136/emermed-2019-209324 | Snakebite not disaggregated; no snakebite-specific pre-hospital outcome data |
| FT13 | Systematic review of human poisoning in Myanmar | 10.3390/ijerph18073576 | Systematic review; no original pediatric snakebite data; secondary source |
| FT14 | Myocarditis complicating viper snake bite in a child | 10.22506/ti/2017/v24/i2/162427 | Single case report — excluded by design |
| FT15 | Amputation of a limb secondary to snakebite in a child (France) | 10.1016/j.arcped.2016.12.013 | Single case report — excluded by design |
| FT16 | Retrospective review of rattlesnake bites in 100 children | 10.23736/S0026-4946.16.04226-2 | In-hospital focus; minimal pre-hospital data extracted; possible duplicate cohort |
| FT17 | Venomous snakebites in children in southern Croatia | 10.1016/j.toxicon.2016.01.057 | In-hospital management only; no pre-hospital or first-aid data |
| FT18 | Hyperglycemia as a risk factor in children after European viper bites | 10.3109/15563650.2015.1113542 | In-hospital clinical study; no first-aid or pre-hospital data |
| FT19 | An epidemiological and clinical study of snake-bites in childhood (Australia 1989) | 10.5694/j.1326-5377.1989.tb136764.x | Pre-hospital data absent; in-hospital management; older study with inadequate methodology |
| FT20 | Envenomation in children (historical review) | Not available | Unable to retrieve full text; no DOI; insufficient data |
| FT21 | Venomous snakebite in two children | Not available | Case reports (n=2); excluded by design; no DOI |
| FT22 | Impact of first aid training in management of snake bite victims in Madi valley | Not available | Unable to retrieve full text; no DOI; insufficient methodological detail |
| FT23 | Controversies in treatment of pediatric crotalinae snake envenomation (review) | 10.1016/S1522-8401(01)90005-9 | Review/guideline; no original pre-hospital data |
| FT24 | Diagnosis and treatment of confirmed and suspected snake bite: 46 paediatric cases (1992) | 10.5694/j.1326-5377.1992.tb139749.x | In-hospital management focus; no first-aid/pre-hospital data |
| FT25 | Suspected snakebite in children: 156 patients over 10 years (Australia) | 10.5694/j.1326-5377.1996.tb122122.x | In-hospital retrospective; insufficient pre-hospital data; duplicate concern with other cohort |
| FT26 | Coral snake bites and envenomation in children: case series | 10.1097/PEC.0000000000000109 | Case series; primarily in-hospital; insufficient pre-hospital detail |
| FT27 | Clinical profile of snake bite in children (Eastern India, hospital-based) — duplicate | Not available | Duplicate cohort with Pattanaik 2023 (included); same institutional cohort |
| FT28 | Snake bites at Fremantle Hospital (10 years) | 10.5694/j.1326-5377.1990.tb126315.x | Not pediatric-specific; adult mixed; no pre-hospital data |
| FT29 | Crotalidae envenomation in children (1993) | 10.1097/00000637-199308000-00009 | In-hospital management only; no pre-hospital outcomes |
| FT30 | Risk factors for high-grade envenomations after French viper bites in children | 10.1097/PEC.0b013e31825cfd66 | In-hospital risk factors; no pre-hospital first-aid data |
| FT31 | Transporting children with toxicological emergencies | 10.1111/1742-6723.12221 | Not snakebite-specific; general toxicological transport; no original data |
| FT32 | Controversies and hazards in treatment of pit viper bites (1979) | 10.1097/00007611-197908000-00002 | Historical review/opinion; no original pediatric data |
| FT33 | Snake Bite in Children: Five Year Study from South-East Queensland (1978) | 10.1111/j.1440-1754.1978.tb02993.x | In-hospital management; no pre-hospital data; methodological detail insufficient |
| FT34 | Envenomation by Northern Blacktail rattlesnake: case report (USA) | 10.1097/01.pec.0000150989.03981.06 | Single case report — excluded by design |
| FT35 | Venomous spiders, snakes, and scorpions in the United States (2009 review) | 10.3928/00904481-20090401-07 | General review; not pediatric-specific; no original pre-hospital data |
| FT36 | Snake bites and scorpion sting (chapter/review) | Not available | Review/editorial; no original pediatric snakebite data; unable to retrieve |
| FT37 | Snakebite: Action stat! (1987 editorial) | 10.1097/00152193-198706000-00016 | Editorial — excluded by design; no original data |
| FT38 | An epidemiological study of snake-bite in childhood (Australia 1989) | 10.5694/j.1326-5377.1989.tb136764.x | Duplicate entry; same as FT19 — in-hospital data only; insufficient pre-hospital information |
| FT39 | Clinical aspects and management of snake bites in emergency department (Turkey) | 10.4328/JCAM.2253 | Not pediatric-specific; adult emergency department study |
| FT40 | Snakebite deaths in Australia 1992-1994 | 10.5694/j.1326-5377.1995.tb124770.x | Not pediatric-specific; adult national epidemiology; no pre-hospital component |
| FT41 | Venomous snake bite: Current concepts of treatment (review) | Not available | Review only; no original pediatric data; excluded by design |
| FT42 | North American snake envenomation: Diagnosis, treatment, and management (review) | 10.1016/j.emc.2004.01.007 | Review; no original pediatric data; in-hospital focus |

*Note: 9 reports sought for full-text review could not be retrieved. These were recorded as 'not retrieved' in the PRISMA flow diagram and excluded accordingly. The exclusion categories at full-text stage were: not pediatric-specific (n=17); no first-aid/pre-hospital care data (n=13); wrong study design (n=7); insufficient data quality/unclear methodology (n=3); duplicate data from the same cohort (n=2).*
