# Supplementary material for: First-aid, pre-hospital care, and harmful indigenous practices in pediatric snakebite envenomation: A systematic review of global evidence from 1973 to 2025
Source: PLoS Negl Trop Dis. 2026 Jul 28;20(7):e0014508. doi: 10.1371/journal.pntd.0014508 (PMC13411870; doi:10.1371/journal.pntd.0014508)
Supplement: S2 Table — (DOCX) [file pntd.0014508.s003.docx]

**S2 Table:** Comprehensive data extraction table detailing study characteristics, extracted variables, names of data extractors, dates of extraction, eligibility confirmation, and notation of any data obtained via author correspondence.

Data were extracted independently by two reviewers (NG = Nayana Gunathilaka; ID = Iranga Dilshan) using a standardized, pilot-tested Excel extraction form. Extraction was completed in January 2026. Discrepancies were resolved through discussion. Where data were unclear, attempts were made to contact study authors (not always successful for older publications). No data in this table were obtained via formal author correspondence except where specifically noted.

*Abbreviations: FA = first aid; AKI = acute kidney injury; HCP = healthcare provider; NOS = Newcastle–Ottawa Scale; JBI = Joanna Briggs Institute; CASP = Critical Appraisal Skills Programme; NTD = neglected tropical disease; PICU = pediatric intensive care unit; OR = odds ratio; NS = not specified. Eligibility confirmed = both reviewers agreed study met all inclusion criteria.*

| **No.** | **Author(s), Year** | **Country** | **Extractors (Initials)** | **Extraction Date** | **Eligibility Confirmed** | **Study Design** | **Sample Size** | **Age Group** | **Setting / Region** | **Snake Species / Type** | **Pre-hospital / FA Variables Assessed** | **Primary Outcomes** | **Key Findings (Pre-hospital)** | **Risk of Bias Tool** | **Author Correspondence** | **Data Gaps Noted** |
| --- | --- | --- | --- | --- | --- | --- | --- | --- | --- | --- | --- | --- | --- | --- | --- | --- |
| 1 | Cristino et al., 2025 | Brazil | NG, ID | Jan 2026 | Yes | Qualitative thematic drawing-and-story | 30 | 6-12 years | Brazilian Amazon | Trematochrysis (coral/lancehead) | First-aid, traditional healers, therapeutic itineraries | Delay to hospital, healer consultation rate | Median delay 8 hrs; 63% saw trad. healer; 47% herbal poultice | NOS N/A; CASP | Not required | No |
| 2 | Dayasiri et al., 2025a | Sri Lanka | NG, ID | Jan 2026 | Yes | Cross-sectional survey | 462 | 0-18 years | Rural Sri Lanka | Naja naja, D. russelii, Hypnale spp. | Tourniquet, immobilization, incision, traditional medicine, health-seeking | Inappropriate FA prevalence, delay >6 hrs | 78% tourniquet; 82% inappropriate FA; 45% delay >6 hrs | NOS 7/9 | Not required | No |
| 3 | Dayasiri et al., 2025b | Sri Lanka | NG, ID | Jan 2026 | Yes | Cross-sectional survey (HCPs) | 156 HCPs | Adults managing pediatric cases | Rural Sri Lanka | Multiple | Training needs, knowledge gaps | Inadequate training prevalence | 67% HCPs inadequate training; gap in pre-hospital triage | NOS 6/9 | Not required | No |
| 4 | Dayasiri et al., 2025c | Sri Lanka | NG, ID | Jan 2026 | Yes | Qualitative interviews | 28 physicians | Adults managing pediatric cases | Rural Sri Lanka | Multiple | Physician perspectives on pre-hospital practices, challenges | Tourniquet complications, management challenges | 34% severe cases had tourniquet complications | CASP | Not required | No |
| 5 | Dayasiri et al., 2025d | Sri Lanka | NG, ID | Jan 2026 | Yes | Cross-sectional household survey | 384 parents | Children 0-15 years | Snakebite hotspots, rural Sri Lanka | Multiple | Preventive practices, parental knowledge/attitudes | Parental first-aid knowledge, appropriate FA identification | 18% parents identified appropriate FA; 41% belief in trad. healer | NOS 7/9 | Not required | No |
| 6 | Dayasiri et al., 2025e | Sri Lanka | NG, ID | Jan 2026 | Yes | Mixed-methods | 420 parents | Children 0-18 years | Rural Sri Lanka | Multiple | Parental beliefs, traditional practices, emergency response | Fear/tradition impact on care delay | 52% trad. healer; 38% spiritual beliefs; 64% delayed care | CASP / NOS | Not required | No |
| 7 | Oliveira et al., 2023 | Brazil | NG, ID | Jan 2026 | Yes | Retrospective cohort | 345 | 0-15 years | Hospital, Brazil | Bothrops spp., Crotalus spp. | Tourniquet, incision, traditional remedies, electric shock, time to hospital | Complications, tourniquet OR | 45% tourniquet; OR 2.8 local complications; 23% incision; 31% traditional remedies | NOS 7/9 | Not required | No |
| 8 | Buitendag et al., 2021b | South Africa | NG, ID | Jan 2026 | Yes | Comparative cohort | 156 (78 pediatric) | 0-16 vs adults | North Eastern South Africa | Cytotoxic spp. | Tourniquet, traditional medicine, pre-hospital delay | Mortality, outcomes vs adults | 38% tourniquet; delay >4 hrs associated with mortality; worse outcomes in children | NOS 6/9 | Not required | No |
| 9 | Suryanarayana et al., 2020 | India | NG, ID | Jan 2026 | Yes | Prospective cohort | 167 | 1-15 years | Tertiary care, India | Naja naja, D. russelii, Bungarus spp. | First-aid practices, time to hospital, risk factors for poor outcomes | Poor outcome predictors | 62% tourniquet; 34% incision; OR 3.4 for delay >6 hrs | NOS 7/9 | Not required | No |
| 10 | Geyt et al., 2020 | Multi-country | NG, ID | Jan 2026 | Yes | Narrative review with cases | N/A (review) | Pediatric focus | Global | Multiple | WHO guidelines, tourniquet, immobilization, rapid transport | Harmful practice identification | Tourniquet and incision identified as harmful; recommended abandonment | JBI | Not required | No |
| 11 | Nduagubam et al., 2020 | Nigeria | NG, ID | Jan 2026 | Yes | Cross-sectional hospital-based | 98 | 2-15 years | Nigeria | Echis spp., others | Comparison of practices with WHO guidelines | WHO-compliant care rate | 89% had ≥1 harmful FA; 71% tourniquet; 58% incision; 0% WHO-recommended care | NOS 6/9 | Not required | No |
| 12 | Variawa et al., 2020 | South Africa | NG, ID | Jan 2026 | Yes | Prospective review | 89 | 0-13 years | South Africa | Cytotoxic (Bitis spp.) | Pre-hospital interventions, tourniquet complications | Compartment syndrome rate | 41% tourniquet; 12% compartment syndrome from tourniquet | NOS 7/9 | Not required | No |
| 13 | Giri et al., 2020 | India | NG, ID | Jan 2026 | Yes | Prospective observational PICU | 52 | 1-12 years | PICU, India | Multiple | First-aid at home, time to hospital, PICU outcomes | Mortality, PICU admission predictors | 11.5% mortality; 67% tourniquet; delay and FA associated with PICU admission | NOS 7/9 | Not required | No |
| 14 | Sood et al., 2020 | India | NG, ID | Jan 2026 | Yes | Retrospective cohort | 134 | 1-15 years | Tertiary care, Himachal Pradesh | Multiple | Epidemiology, first-aid, clinical profile | Local complications, tourniquet association | 58% tourniquet; 42% traditional remedies; higher local complications with tourniquet | NOS 6/9 | Not required | No |
| 15 | Pandey et al., 2020 | Nepal | NG, ID | Jan 2026 | Yes | Cross-sectional school survey | 422 students | 10-18 years | Schools, Nepal | Multiple | Student perceptions, knowledge of snakes, first-aid knowledge | Knowledge scores, beliefs | 67% had traditional beliefs; 78% inadequate FA knowledge; educational intervention improved scores | NOS 6/9 | Not required | No |
| 16 | Bush & Kinlaw, 2015 | USA | NG, ID | Jan 2026 | Yes | Case report with management review | 1 | 8 years | USA | Crotalus spp. | Tight tourniquet complications, surgical management | Limb ischemia outcome | Severe limb ischemia from 24-hour tight tourniquet; emergency surgery required | JBI | Not required | No |
| 17 | Sankar et al., 2013 | India | NG, ID | Jan 2026 | Yes | Prospective observational | 212 | 1-12 years | Tertiary hospital, India | Multiple | First-aid, time to hospital, outcome predictors | Severity odds per hour delay | 54% tourniquet; each hour delay: OR 1.4 for severity; tourniquet associated with complications | NOS 8/9 | Not required | No |
| 18 | Mars et al., 1991 | South Africa | NG, ID | Jan 2026 | Yes | Case series with compartment pressure monitoring | 8 | 4-13 years | South Africa | Cytotoxic spp. | Tourniquet use, compartment syndrome, surgical intervention | Compartment pressure, fasciotomy rate | All had tourniquet; direct pressure monitoring; 3/8 required fasciotomy | JBI | Not required | No |
| 19 | Tadros et al., 2022 | USA | NG, ID | Jan 2026 | Yes | Retrospective ED database analysis | 8,567 | 0-17 years | US emergency departments | Multiple (North American) | ED visit patterns, pre-hospital care utilization | Pre-hospital care patterns, complications | Most received appropriate pre-hospital care; low complication rate; limited FA data in records | NOS 7/9 | Not required | No |
| 20 | Schulte et al., 2016 | USA | NG, ID | Jan 2026 | Yes | Retrospective national database | 25,770 | 0-19 years | USA national | Multiple (North American) | Epidemiology, hospitalization, outcomes, FA practices | Mortality, inappropriate FA rates | 0.06% mortality; 2.3% tourniquet; 0.8% incision; rare in high-resource setting | NOS 7/9 | Not required | No |
| 21 | Sanni et al., 2021 | Nigeria | NG, ID | Jan 2026 | Yes | Prospective cohort | 67 | 1-14 years | Nigeria | Echis spp., others | FA, traditional healer use, outcomes | Mortality, traditional healer use rate, delay | 10.4% mortality; 73% traditional healer; 64% incision; mean delay 18 hrs | NOS 7/9 | Not required | No |
| 22 | Pattanaik et al., 2023 | India | NG, ID | Jan 2026 | Yes | Retrospective cohort | 142 | 1-15 years | Eastern India | Multiple | Clinical profile, FA, AKI risk | AKI incidence, FA association | 18% AKI; 61% tourniquet; 44% traditional medicine; pre-hospital delay associated with AKI | NOS 7/9 | Not required | No |
| 23 | Ahmed et al., 2019 | Sudan | NG, ID | Jan 2026 | Yes | Descriptive cross-sectional | 186 | 1-15 years | Eastern Sudan | Multiple African spp. | Traditional practices, clinical presentations, management | Mortality, additional injuries from harmful practices | 8.6% mortality; 68% trad. healer; 34% cauterization; 52% incision; 58% herbal remedies | JBI | Not required | No |
| 24 | Marano et al., 2021 | Italy | NG, ID | Jan 2026 | Yes | Case series | 24 | 2-14 years | Italy | Vipera aspis, V. berus | European viper bites, pre-hospital management, pediatric approach | Local tissue damage, ice application | 25% ice application associated with increased tissue damage; 8% tourniquet | JBI | Not required | No |
| 25 | Levine, 2014 | USA | NG, ID | Jan 2026 | Yes | Clinical review | N/A | Pediatric focus | USA | North American pit vipers | Review of pediatric envenomation management, FA principles | Harmful practices identified | Identified common harmful practices; emphasized FA education need | JBI | Not required | No |
| 26 | Matteucci et al., 2007 | USA | NG, ID | Jan 2026 | Yes | Retrospective cohort | 109 | 0-18 years | USA | Crotalus spp. | Gender differences in bite location, antivenom use | Bite location by sex | Boys: higher upper extremity bites; limited pre-hospital data in study | NOS 5/9 | Not required | No |
| 27 | Marano et al., 2014b | Italy | NG, ID | Jan 2026 | Yes | Retrospective PICU review | 48 | 1-15 years | Rome, Italy | Vipera spp. | Antitoxin use, intensive care management, FA prior to admission | PICU admission predictors | 21% ice application; 65% inadequate immobilization; PICU admission associated with delayed antivenom | JBI | Not required | No |
| 28 | Pivko-Levy et al., 2017 | Israel | NG, ID | Jan 2026 | Yes | Retrospective two-center study | 91 | 0-18 years | Israel | Vipera palaestinae | Antivenom therapy evaluation, pre-hospital practices | Antivenom outcomes, tourniquet trend | 14% tourniquet; declining over study period; good outcomes with appropriate care | NOS 6/9 | Not required | No |
| 29 | Lifshitz et al., 1995 | Israel | NG, ID | Jan 2026 | Yes | Case reports | 2 | 3 and 7 years | Israel | Cerastes vipera | Cerastes vipera envenomation management, FA | Case outcomes | No harmful practices in these cases; rapid transport emphasized | JBI | Not required | No |
| 30 | Narra et al., 2014 | USA | NG, ID | Jan 2026 | Yes | Retrospective resource utilization | 2,148 | 0-18 years | USA | Multiple | Healthcare resource use, hospital admissions | Healthcare costs and utilization | Focus on healthcare resource use; limited pre-hospital data | NOS 6/9 | Not required | No |
| 31 | Cordasco et al., 2001 | USA | NG, ID | Jan 2026 | Yes | Clinical review | N/A | Pediatric focus | USA | Multiple | Air medical transport, treatment protocols, harmful FA review | FA recommendations | Tourniquet, incision, ice, suction recommended against | JBI | Not required | No |
| 32 | Rumore & Heaney, 2018 | Australia | NG, ID | Jan 2026 | Yes | Case report | 1 | 3 years | Australia | Notechis scutatus (tiger snake) | Tiger snake bite, severe neuropathy/myopathy, pressure immobilization | Clinical outcome with appropriate FA | Pressure immobilization applied appropriately; prolonged complications despite optimal management | JBI | Not required | No |
| 33 | Offerman et al., 2002 | USA | NG, ID | Jan 2026 | Yes | Prospective treatment study | 31 | 1-18 years | USA | Crotalus spp. | Crotaline Fab antivenom efficacy, FA prior to presentation | Antivenom outcomes, pre-hospital FA | 6% tourniquet; good outcomes with appropriate care; Fab antivenom effective in pediatric use | NOS 6/9 | Not required | No |
| 34 | Chatterjee et al., 2022 | India | NG, ID | Jan 2026 | Yes | Hospital-based cross-sectional | 78 | 1-14 years | Eastern India | Multiple | FA practices, clinical profile | Inappropriate FA prevalence | 87% inappropriate FA; 69% tourniquet; 51% traditional medicine; 37% incision | NOS 6/9 | Not required | No |
| 35 | Kumar et al., 2024 | India | NG, ID | Jan 2026 | Yes | Retrospective cohort | 198 | 1-15 years | Bihar, India | Multiple | Epidemiology, FA, clinical insights | Delayed presentation rates, healer use | 64% tourniquet; 42% traditional healer; 56% delay >6 hrs | NOS 6/9 | Not required | No |
| 36 | Anil Kumar et al., 2017 | India | NG, ID | Jan 2026 | Yes | Hospital-based study | 156 | 1-15 years | India | Multiple | Clinico-epidemiological profile, FA | Local complications with tourniquet | 56% tourniquet; 34% traditional remedies; higher local complications with tourniquet | NOS 6/9 | Not required | No |
| 37 | Henderson & Dujon, 1973 | Not specified | NG, ID | Jan 2026 | Yes | Retrospective review | 42 | 0-15 years | Hospital (country NS) | Not specified | Early surgical series on pediatric snakebites, FA practices | Historical first-aid education need | Limited FA data; historical perspective; identified need for systematic FA education | JBI | Not required | No |
| 38 | Goto & Feng, 2009 | USA | NG, ID | Jan 2026 | Yes | Retrospective review | 68 | 0-18 years | USA | Crotalus spp. | Crotalidae polyvalent immune FAB treatment, pre-hospital FA | Antivenom outcomes, harmful FA rates | 7% tourniquet; 4% ice; low rates of inappropriate FA; good outcomes | NOS 6/9 | Not required | No |
| 39 | Pandian et al., 2023 | India | NG, ID | Jan 2026 | Yes | Retrospective cohort | 118 | 1-16 years | South India | Multiple | AKI risk factors, pre-hospital delay, tourniquet | AKI incidence, tourniquet as independent risk factor | 22% AKI; 59% tourniquet; pre-hospital delay and tourniquet both independent AKI risk factors | NOS 7/9 | Not required | No |
| 40 | Rashad, 2019 | Saudi Arabia | NG, ID | Jan 2026 | Yes | Descriptive study | 85 | 2-16 years | Al-Baha, Saudi Arabia | Multiple | Epidemiology, presentations, prevention, FA practices | Community education need | 31% tourniquet; 24% traditional remedies; 8% cauterization; community education recommended | JBI | Not required | No |
| 41 | De Albuquerque et al., 2014 | Brazil | NG, ID | Jan 2026 | Yes | Cross-sectional school study | 156 students | 10-17 years | Brazil | Multiple | First-aid knowledge assessment in students | Pre/post-intervention knowledge | 82% pre-intervention inadequate FA knowledge; educational intervention improved scores significantly | NOS 6/9 | Not required | No |
| 42 | Hussein & Elrewany, 2023 | Egypt | NG, ID | Jan 2026 | Yes | Interventional (pre-post) | 240 students | 10-14 years | Egypt | Multiple | Effectiveness of first-aid educational program | Knowledge improvement, sustainability | Baseline 89% inadequate knowledge; 76% harmful practice beliefs; significant improvement post-intervention; sustained at 3 months | NOS 7/9 | Not required | No |
| 43 | Halbert et al., 2015 | Myanmar | NG, ID | Jan 2026 | Yes | PICU service review (12-month) | 12-month data | 0-15 years | Myanmar PICU | Multiple | PICU patterns, service development, FA education need | PICU admission rates | High PICU admissions for snakebite; need for FA education identified | JBI | Not required | No |
| 44 | Harbi, 1999 | Saudi Arabia | NG, ID | Jan 2026 | Yes | Comparative study (children vs adults) | 67 (39 children) | Children vs adults | South Western Saudi Arabia | Multiple | Epidemiology, clinical differences by age, FA practices | Age group differences in FA | 43% tourniquet; 28% traditional remedies; children had higher rates of inappropriate FA than adults | NOS 6/9 | Not required | No |
