# Supplementary material for: First-aid, pre-hospital care, and harmful indigenous practices in pediatric snakebite envenomation: A systematic review of global evidence from 1973 to 2025
Source: PLoS Negl Trop Dis. 2026 Jul 28;20(7):e0014508. doi: 10.1371/journal.pntd.0014508 (PMC13411870; doi:10.1371/journal.pntd.0014508)
Supplement: S3 Table — (DOCX) [file pntd.0014508.s004.docx]

**S3 Table:** Completed risk of bias assessments for each included study using the Newcastle–Ottawa Scale (observational studies), Joanna Briggs Institute Critical Appraisal Checklist (case series/descriptive studies), and CASP Qualitative Checklist (qualitative studies), with domain-level responses provided

Risk of bias was assessed independently by two reviewers. Disagreements were resolved by discussion or third-reviewer arbitration. Tools used: Newcastle–Ottawa Scale (NOS) for observational studies (cohort, cross-sectional, case-control); JBI Critical Appraisal Checklist for Case Series/Descriptive Studies; CASP Qualitative Checklist for qualitative studies. Quality results informed narrative synthesis but were not used as exclusion criteria.

**Part A: Newcastle–Ottawa Scale; Observational Studies**

*NOS domains: Selection (max 4 stars), Comparability (max 2 stars), Outcome/Exposure (max 3 stars). Total /9 stars. Adapted NOS applied for cross-sectional studies. Risk classification: Low = 7-9; Moderate = 5-6; High = <5.*

| **No.** | **Author(s), Year** | **Study Design** | **Selection (0–4)** | **Comparability (0–2)** | **Outcome / Exposure (0–3)** | **Total Score** | **Risk of Bias** |
| --- | --- | --- | --- | --- | --- | --- | --- |
| 2 | Dayasiri et al., 2025a | Cross-sectional | 3 | 1 | 2 | **6/9** | **Moderate** |
| 3 | Dayasiri et al., 2025b | Cross-sectional | 3 | 1 | 2 | **6/9** | **Moderate** |
| 5 | Dayasiri et al., 2025d | Cross-sectional | 3 | 2 | 2 | **7/9** | **Low** |
| 7 | Oliveira et al., 2023 | Retrospective cohort | 4 | 2 | 2 | **7/9 (adapted)** | **Low** |
| 8 | Buitendag et al., 2021b | Comparative cohort | 3 | 1 | 2 | **6/9** | **Moderate** |
| 9 | Suryanarayana et al., 2020 | Prospective cohort | 4 | 2 | 2 | **7/9 (adapted)** | **Low** |
| 11 | Nduagubam et al., 2020 | Cross-sectional | 3 | 1 | 2 | **6/9** | **Moderate** |
| 12 | Variawa et al., 2020 | Prospective review | 4 | 1 | 2 | **7/9 (adapted)** | **Low** |
| 13 | Giri et al., 2020 | Prospective observational | 4 | 2 | 2 | **7/9 (adapted)** | **Low** |
| 14 | Sood et al., 2020 | Retrospective cohort | 3 | 1 | 2 | **6/9** | **Moderate** |
| 15 | Pandey et al., 2020 | Cross-sectional school survey | 3 | 1 | 2 | **6/9** | **Moderate** |
| 17 | Sankar et al., 2013 | Prospective observational | 4 | 2 | 3 | **8/9 (adapted)** | **Low** |
| 19 | Tadros et al., 2022 | Retrospective database | 4 | 1 | 2 | **7/9 (adapted)** | **Low** |
| 20 | Schulte et al., 2016 | Retrospective national database | 4 | 2 | 2 | **7/9 (adapted)** | **Low** |
| 21 | Sanni et al., 2021 | Prospective cohort | 4 | 1 | 2 | **7/9 (adapted)** | **Low** |
| 22 | Pattanaik et al., 2023 | Retrospective cohort | 4 | 1 | 2 | **7/9 (adapted)** | **Low** |
| 26 | Matteucci et al., 2007 | Retrospective cohort | 3 | 1 | 1 | **5/9** | **Moderate-high** |
| 28 | Pivko-Levy et al., 2017 | Retrospective two-center | 3 | 1 | 2 | **6/9** | **Moderate** |
| 30 | Narra et al., 2014 | Retrospective | 3 | 1 | 2 | **6/9** | **Moderate** |
| 33 | Offerman et al., 2002 | Prospective treatment | 4 | 1 | 2 | **6/9 (adapted)** | **Moderate** |
| 34 | Chatterjee et al., 2022 | Cross-sectional | 3 | 1 | 2 | **6/9** | **Moderate** |
| 35 | Kumar et al., 2024 | Retrospective cohort | 3 | 1 | 2 | **6/9** | **Moderate** |
| 36 | Anil Kumar et al., 2017 | Hospital-based | 3 | 1 | 2 | **6/9** | **Moderate** |
| 38 | Goto & Feng, 2009 | Retrospective | 3 | 1 | 2 | **6/9** | **Moderate** |
| 39 | Pandian et al., 2023 | Retrospective cohort | 4 | 1 | 2 | **7/9 (adapted)** | **Low** |
| 41 | De Albuquerque et al., 2014 | Cross-sectional | 3 | 1 | 2 | **6/9** | **Moderate** |
| 42 | Hussein & Elrewany, 2023 | Interventional pre-post | 3 | 1 | 2 | **7/9 (adapted)** | **Low** |
| 44 | Harbi, 1999 | Comparative | 3 | 1 | 2 | **6/9** | **Moderate** |

**Part B: JBI Critical Appraisal Checklist; Case Series & Descriptive Studies**

*JBI questions (abbreviated): Q1=Clear criteria for inclusion; Q2=Condition measured reliably; Q3=Valid methods used; Q4=Consecutive or clearly defined inclusion; Q5=Complete data; Q6=Outcomes measured objectively; Q7=Follow-up period appropriate; Q8=Outcomes reported completely; Q9=Statistical analysis appropriate (where applicable). Responses: Yes / No / Unclear / N/A. Risk: Low ≥6/8; Moderate 4-5/8; High <4/8.*

| **No.** | **Author(s), Year** | **Study Type** | **Q1** | **Q2** | **Q3** | **Q4** | **Q5** | **Q6** | **Q7** | **Q8** | **Q9** | **Total** | **Risk of Bias** |
| --- | --- | --- | --- | --- | --- | --- | --- | --- | --- | --- | --- | --- | --- |
| 10 | Geyt et al., 2020 | Narrative review/cases | Yes | Yes | Yes | Yes | Unclear | Yes | Yes | Yes | N/A | **7/8 applicable** | **Low** |
| 16 | Bush & Kinlaw, 2015 | Case report | Yes | Yes | Yes | Yes | Yes | Yes | Unclear | Unclear | N/A | **6/8** | **Moderate** |
| 18 | Mars et al., 1991 | Case series | Yes | Yes | Yes | Yes | Yes | Unclear | Unclear | Yes | N/A | **6/8** | **Moderate** |
| 23 | Ahmed et al., 2019 | Descriptive cross-sectional | Yes | Yes | Yes | Yes | Unclear | Yes | Yes | Unclear | N/A | **6/8** | **Moderate** |
| 24 | Marano et al., 2021 | Case series | Yes | Yes | Yes | Yes | Unclear | Yes | Yes | Yes | N/A | **7/8** | **Low** |
| 25 | Levine, 2014 | Clinical review | Yes | Yes | Yes | Unclear | Unclear | Yes | Yes | Unclear | N/A | **5/8** | **Moderate** |
| 27 | Marano et al., 2014b | PICU case review | Yes | Yes | Yes | Yes | Unclear | Yes | Yes | Yes | N/A | **7/8** | **Low** |
| 29 | Lifshitz et al., 1995 | Case reports | Yes | Yes | Yes | Yes | Unclear | Unclear | Unclear | Unclear | N/A | **4/8** | **Moderate-high** |
| 31 | Cordasco et al., 2001 | Clinical review | Yes | Yes | Unclear | Yes | Unclear | Yes | Yes | Unclear | N/A | **5/8** | **Moderate** |
| 32 | Rumore & Heaney, 2018 | Case report | Yes | Yes | Yes | Yes | Yes | Yes | Unclear | Unclear | N/A | **6/8** | **Moderate** |
| 37 | Henderson & Dujon, 1973 | Retrospective review | Yes | Unclear | Yes | Yes | Unclear | Unclear | Unclear | Unclear | N/A | **4/8** | **Moderate-high** |
| 40 | Rashad, 2019 | Descriptive study | Yes | Yes | Yes | Yes | Unclear | Yes | Yes | Unclear | N/A | **6/8** | **Moderate** |
| 43 | Halbert et al., 2015 | PICU service review | Yes | Yes | Yes | Unclear | Unclear | Yes | Yes | Unclear | N/A | **5/8** | **Moderate** |

**Part C: CASP Qualitative Checklist; Qualitative Studies**

*CASP questions (abbreviated): Q1=Clear aims; Q2=Qualitative methodology appropriate; Q3=Research design appropriate; Q4=Recruitment strategy appropriate; Q5=Data collection appropriate; Q6=Reflexivity considered; Q7=Ethical issues considered; Q8=Data analysis rigorous; Q9=Findings clearly stated; Q10=Research valuable. Responses: Yes / No / Can't tell. Risk: Low = ≥8 Yes; Moderate = 5-7 Yes; High = <5 Yes.*

| **No.** | **Author(s), Year** | **Q1** | **Q2** | **Q3** | **Q4** | **Q5** | **Q6** | **Q7** | **Q8** | **Q9** | **Q10** | **Summary Assessment** | **Risk of Bias** |
| --- | --- | --- | --- | --- | --- | --- | --- | --- | --- | --- | --- | --- | --- |
| 1 | Cristino et al., 2025 | Yes | Yes | Yes | Yes | Yes | Yes | Yes | Yes | Yes | Yes | **Meets all CASP criteria** | **Low** |
| 4 | Dayasiri et al., 2025c | Yes | Yes | Yes | Yes | Yes | Yes | Yes | Yes | Yes | Yes | **Meets all CASP criteria** | **Low** |
| 6 | Dayasiri et al., 2025e | Yes | Yes | Yes | Yes | Yes | Yes | Yes | Yes | Can't tell | Yes | **Strong qualitative rigour** | **Low** |

*Overall quality summary: Of the 44 included studies, 15 were rated low risk of bias, 26 moderate risk, and 3 moderate-high risk. No study was excluded solely on the basis of quality. The moderate-to-high risk studies were predominantly older case series and case reports with limited methodological detail; their findings were interpreted with appropriate caution during narrative synthesis.*

Following references were added.

**References**

1. Wells GA, Shea B, O’Connell D, Peterson J, Welch V, Losos M, et al. The Newcastle-Ottawa Scale (NOS) for assessing the quality of nonrandomised studies in meta-analyses. Ottawa Hospital Research Institute; 2000. Available from: http://www.ohri.ca/programs/clinical_epidemiology/oxford.asp

2. Joanna Briggs Institute. JBI Critical Appraisal Tools. Adelaide: JBI; 2020. Available from: https://jbi.global/critical-appraisal-tools

3. Critical Appraisal Skills Programme (CASP). CASP Qualitative Studies Checklist. Oxford: CASP; 2018. Available from: https://casp-uk.net/casp-tools-checklists/
